# MGMT, NUPR1, NDRG2, and GLI1 Gene Promoter Methylation in Glioblastoma Tissues and Association with Clinical Characteristics and Therapeutic Outcomes

**DOI:** 10.3390/ijms27020763

**Published:** 2026-01-12

**Authors:** Mariam M. Gabr, Sherihan G. AbdelHamid, Lobna R. Ezz El Arab, Menha Swellam, Nadia M. Hamdy

**Affiliations:** 1 Ain Shams University Hospitals, Abassia, Cairo 11566, Egypt; 2 Biochemistry and Molecular Biology Department, Faculty of Pharmacy, Ain Shams University, Abassia, Cairo 11566, Egypt; 3 Clinical Oncology Department, Faculty of Medicine, Ain Shams University, Abassia, Cairo 11566, Egypt; 4 Biochemistry Department, Biotechnology Research Institute, High Throughput Molecular and Genetic Laboratory, Central Laboratories Network and the Center for Excellences for Advanced Sciences, National Research Centre, Dokki, Giza 12622, Egypt

**Keywords:** glioblastoma (GBM), Epigenetics, DNA methylation, in silico, MGMT, NUPR1, NDRG2, GLI1, bioinformatics

## Abstract

Glioblastoma (GBM) is the most prevalent and devastating form of primary brain tumors in adults, with dismal survival despite advancements in treatment modalities. The current study sought to develop clinically significant prognostic models for GBM patients by comprehensively profiling MGMT, NUPR1, NDRG2, and GLI1 gene promoter methylation in GBM tissues vs. non-neurooncological disease (NND) and their association with clinical characteristics and therapeutic outcome. This was further evaluated by in silico functional enrichment analysis. NUPR1, NDRG2, and GLI1 gene promoter methylation were significant epigenetic discriminators between GBM and NND. However, NDRG2 methylation was the sole independent predictor for neoplastic lesions (OR = 1.71, 95% CI [1.25–3.57], *p* = 0.028). Multivariable Cox regression analysis revealed that NUPR1 promoter hypermethylation was significantly correlated with a lower risk of mortality (HR = 0.96, 95% CI [0.96–0.99], *p* = 0.002), while multiple tumor sites were linked to an increased risk of mortality in the univariate model (HR = 4.44, 95% CI [1.42–13.88], *p* = 0.01). A heatmap correlation matrix identified a robust positive correlation among the MGMT and NUPR1 methylation status (r = 0.93, *p* < 0.001). NUPR1 and MGMT promoter hypermethylation was associated with a favorable response to temozolomide therapy. Patients with NUPR1 and MGMT hypermethylation exhibited extended OS and PFS compared to those with hypomethylation levels, whereas GLI1 and NDRG2 hypermethylation were linked to shorter PFS. In conclusion, the multi-faceted epigenetic panel adopted in the current study captures different aspects of GBM biology and moves towards a more comprehensive model that reflects the molecular heterogeneity of GBM as insights for personalized therapy.

## 1. Introduction

Glioblastoma (GBM) is a highly prevalent and lethal primary brain malignancy in adults, comprising nearly 60% of all primary brain malignancies [1] and more than 14% of all malignant central nervous system (CNS) malignancies [2,3]. Necrosis and microvascular proliferation (MVP) are histopathologic characteristics that define grade 4 glioma [4]. GBM usually appears at the age of 64 years, but it can also appear at any age [5], with a slightly higher incidence in males than females (1.6:1) [6], and it is one of the cancer types that is gender-linked [7]. GBMs are categorized as de novo or primary when they emerge without an identifiable precursor and as secondary when a low-grade progressive evolves into a GBM [8]. The majority of GBMs are primary, where the patients are typically older and have a worse prognosis in comparison to those with secondary GBMs [3]. Differences in gene expression as well as molecular modifications between primary and secondary gliomas have been identified [9]. Mutations in the phosphatase and tensin homolog (PTEN) gene, upregulation of the epidermal growth factor receptor (EGFR), and deletion of chromosome 10q are among the genetic alterations that are prevalent in primary GBM [10], while secondary GBM is frequently associated with chromosome 19q deletion, Isocitrate Dehydrogenase 1 (IDH1) mutation, and Tumor Protein p53 (P53) mutation [9,11].

Maximal safe surgical resection is the recommended course of procedure, followed by radiotherapy in conjunction with the oral alkylating chemotherapeutic agent temozolomide (TMZ) [12]. The radical excision of the original tumor mass is not curative due to its high invasiveness [3]; therefore, sometimes infiltrating tumor cells remain in the surrounding brain tissue, leading to progression or recurrence of the disease [13].

Recent studies have shown that GBM progression is strongly linked to various epigenetic phenomena, such as histone modulations, DNA methylation, chromatin remodeling, and aberrant expression of microRNAs [14], which work in tandem with genetic alterations to drive glioma features [15]. Furthermore, due to the reversibility of these epigenetic modifications, the genes and proteins that control these alterations have emerged as novel targets for GBM treatment [16]. The mechanisms that play crucial roles in GBM lethality and resistance have been linked to epigenetic changes [17]. As a result, it is critical to comprehend the epigenetic alterations, such as DNA methylation, that have been linked to GBM resistance. The role of DNA methylation in glioblastoma progression is well-established [18].

In GBM, the promoter methylation status of the enzyme O6-methylguanine (O6-MeG)-DNA methyltransferase (MGMT) is a prognostic and predictive biomarker [19]. MGMT protein blocks the therapeutic effect of alkylating chemotherapeutic drugs, such as TMZ [20]. A growing body of evidence suggests that patients with MGMT silencing through DNA hypermethylation respond better to TMZ and have longer overall survival (OS) [21]. The N-myc downregulated gene (NDRG) family is a collection of genes that predominantly limit tumor growth [22]. N-Myc Downstream-Regulated Gene 2 (NDRG2), which is located on chromosome 14q11.2 [23], has been linked to decreased GBM progression and better prognosis [24]. Glioblastomas show significant alterations in NDRG2 expression [25]. The main molecular mechanism responsible for the frequent transcriptional downregulation of NDRG2 expression observed in primary glioblastoma appears to be aberrant promoter methylation [26].

NUclear PRotein 1 (NUPR1) is a nuclear protein that acts as a transcriptional coactivator or corepressor depending on the context [27]. It has been implicated in various cellular processes such as inflammation, apoptosis, autophagy, and DNA damage response [28]. In GBM, NUPR1 has been shown to promote cell proliferation, invasion, and resistance to apoptosis by activating the PI3K/AKT/mTOR pathway [29]. The methylation status of the NUPR1 promoter region controls its expression [30]. Gene silencing is usually caused by hypermethylation of CpG sites, whereas gene activation is usually caused by hypomethylation of CpG sites [31]. Glioma-associated oncogene homolog 1 (GLI1) is a transcription factor (TF) that is involved in embryonic development and tissue homeostasis via mediating the hedgehog (HH) signaling pathway [32]. The HH pathway’s abnormal activation has been linked to a variety of malignancies, including GBM [33]. GLI1 has been demonstrated to increase GBM cell proliferation, survival, angiogenesis, and stemness through regulation of target genes, including B-cell leukemia/lymphoma 2 protein (BCL2), vascular endothelial growth factor (VEGF), and sex-determining region Y-box 2 (SOX2) [34].

Problem. GBM is a devastating brain tumor characterized by its aggressive nature and resistance to conventional therapies [35]. Despite advancements in treatment modalities, the prognosis for those patients remains dismal, with a median OS of fewer than 15 months [36]. The molecular mechanisms underlying the poor prognosis of GBM patients are not fully understood [37], and there is a need to identify more reliable prognostic molecular markers that can aid in personalized therapeutic strategies.

Aim. The purpose of this study was to develop a clinically useful prognostic model for GBM patients to improve the precision of treatment decisions by comprehensively profiling DNA promoter methylation using four candidate genes (MGMT, NUPR1, NDRG2, and GLI1) in GBM patients in comparison to patients with non-neurooncological disease (NND). The selection process was based on a combination of biological relevance, literature evidence, and preliminary bioinformatics screening.

MGMT was selected as the benchmark and cornerstone of our panel, as it is a well-established predictive biomarker in GBM, where promoter methylation has been directly linked to response to TMZ therapy and overall prognosis. Its inclusion allows for a direct comparison of the novel candidates (NUPR1 and GLI1) against a gold standard, thereby calibrating the prognostic and predictive power of our proposed panel.

NUPR1 was selected due to its emerging role as a key stress-induced oncoprotein. Evidence indicates that NUPR1 promotes GBM cell proliferation, invasion, and resistance to apoptosis and chemotherapy (e.g., TMZ) by activating oncogenic pathways, like PI3K/AKT/mTOR, and regulating autophagy. Crucially, its expression is known to be epigenetically regulated. Therefore, we hypothesized that its methylation status could be a novel predictor of therapeutic resistance and poor outcome, serving as a potential counterpoint or complement to MGMT because of its reported role in promoting cell proliferation, invasion, and resistance to apoptosis in GBM. The methylation status of the NUPR1 promoter region is known to control its expression. Gene silencing is typically associated with hypermethylation of CpG sites.

NDRG2 was chosen as a putative tumor suppressor frequently epigenetically silenced in GBM. Numerous studies have documented that NDRG2 promoter hypermethylation leads to its downregulation, which is associated with higher tumor grade, increased aggressiveness, and worse patient prognosis. Investigating its methylation status allows us to explore a mechanism of tumor suppressor inactivation that contributes to gliomagenesis and progression.

GLI1 was included as a central transcriptional effector of the oncogenic Hedgehog (HH) signaling pathway, which is aberrantly activated in GBM and other cancers. GLI1 drives processes critical for GBM aggressiveness, including stemness maintenance, proliferation, angiogenesis, and therapy resistance. While often activated by genetic mutations, its expression can also be modulated epigenetically. We proposed that its methylation status could provide insights into the activation of this critical pathway and serve as a prognostic marker.

Objectives. The goal of the study was to elucidate the clinical potential role of promoter methylation patterns of the MGMT, NUPR1, NDRG2, and GLI1 genes as epigenetic molecular indicator markers for GBM via, first, detecting and comparing the promoter methylation status of the four genes in GBM and NND samples using a quantitative PCR-based method. Second, the best combination of DNA methylation molecular markers that can predict survival and responsiveness to therapy of GBM patients will be identified and validated. Third, a multivariate methylation model that can accurately estimate the clinical outcomes of GBM patients will be developed. In silico bioinformatics analysis will be extensively performed as well. The overarching rationale was to create a multi-faceted epigenetic panel that captures different aspects of GBM biology: DNA repair and chemoresistance (MGMT), stress response and oncogenic signaling (NUPR1), tumor suppression (NDRG2), and developmental pathway activation (GLI1).

## 2. Results

### 2.1. Differential Gene Expression (DGE) of Different Genes from Online Datasets in GBM

**Figure 1:**
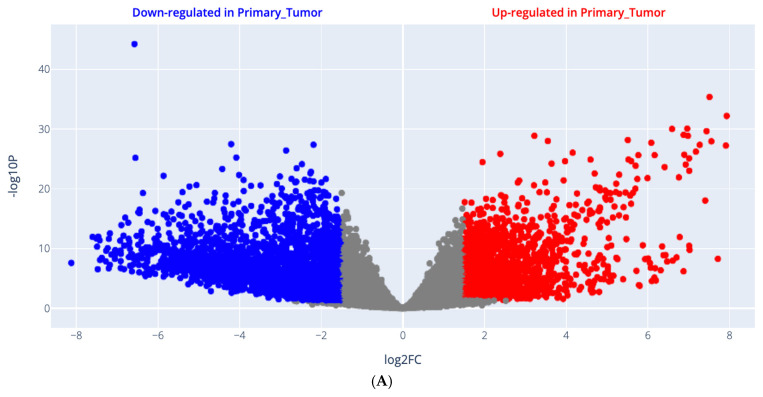

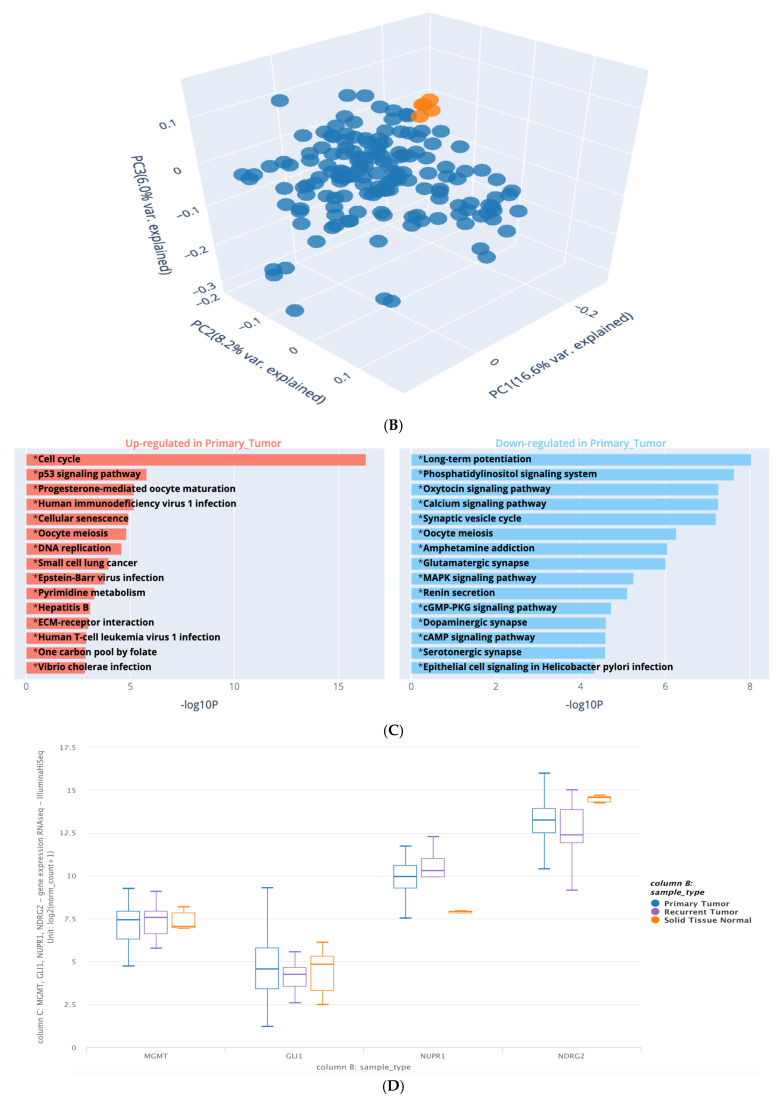

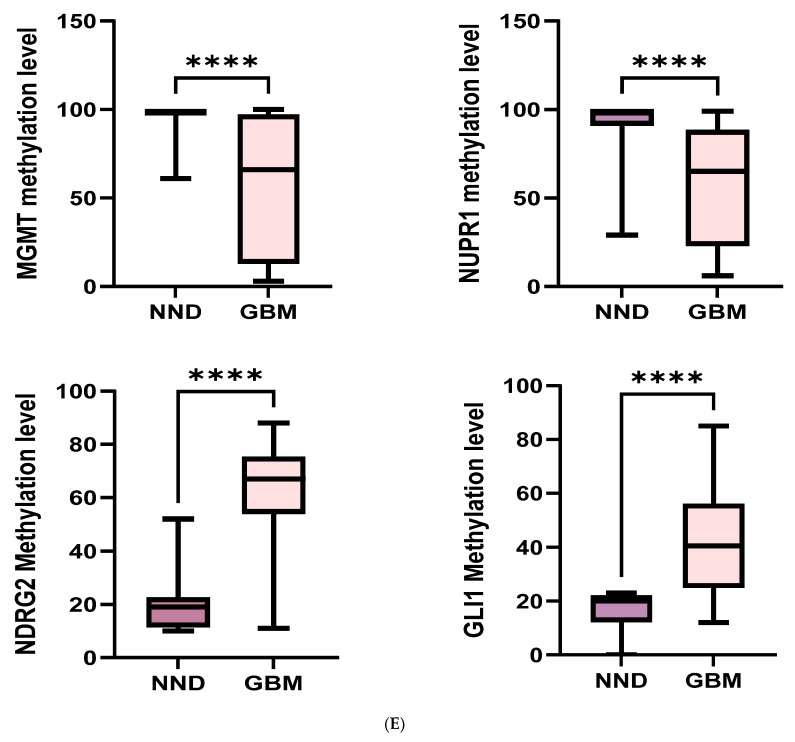
(**A**) Volcano scatter plot for Primary_Tumor vs. Solid_Tissue_Normal displaying the log2 fold changes and statistical significance of each gene calculated by performing a DGE (16,988 genes) analysis. Genes with logFC > 1.5 and *p* value < 0.05 are in red, and genes with logFC < −1.5 and *p* value < 0.05 are in blue. Log-transformed data and base 2 exponentiation are applied. (**B**) A 3D PCA scatter plot for samples using 2500 genes having the largest variance, and each point represents a gene expression sample. Samples with similar gene expression profiles are closer in the three-dimensional space. The normal solid tissue sample group is indicated in orange, and the solid primary tumor is in blue. (**C**) Pathway enrichment analysis, with KEGG pathway Primary_Tumor vs. Solid_Tissue_Normal. (**D**) A comparison of the genes of interest in the Primary_Tumor (*n* = 602) vs. Solid_Tissue_Normal (*n* = 12) in the TCGA glioblastoma (GBM) cohort dataset. (**E**) Different methylation patterns of MGMT, NUPR1, NDRG2, and GLI1 genes among NND (*n* = 20) and GBM (*n* = 58) tested groups; figures were generated using GraphPad Prism software (version 9.5.1), with a Mann–Whitney U test (U) utilized to compare the methylation levels of MGMT, NUPR, NDRG2, and GLI1 genes between NND and GBM groups. The level of significance *p* value was set at 0.05. [NND: non-neurooncological disease, GBM: glioblastoma, MGMT: O6-methylguanine-DNA methyltransferase, NUPR1: Nuclear Protein 1, NDRG2: N-Myc Downstream-Regulated Gene 2, GLI1: glioma-associated oncogene homolog 1; **** *p* value < 0.0001].

Gene expression signatures plotted as a volcano scatter plot of changes in gene expression patterns that arise due to cellular disturbances, such as drug treatments, gene knockdowns, or diseases, are shown in Figure 1A, including all upregulated genes, which are presented in red color, and downregulated genes in GBM are presented in blue color. Moreover, the genes with their log2FC, average expression, and *p* value are attached as a Supplementary Excel File. The 3D scatter plot for PCA results is shown in Figure 1B and is retrieved from UCSC Xena, http://analysis.xenahubs.net/e08c31d726d85e8e343e534eae6c6232245b1768/ (accessed on 27 July 2024).

### 2.2. Enrichment Analysis (EA) Results

Pathway enrichment analysis results are demonstrated in Figure 1C, where the contributing pathways in LIHC pathogenesis are arranged in descending order, with upregulated pathways shown in red and downregulated pathways shown in blue. GO enrichment analysis results are demonstrated in Figure 1D, where the contributing biological processes in GBM pathogenesis are arranged in descending order, while the upregulated biological processes are shown in red and the downregulated biological processes are shown in blue.

### 2.3. Demographic and Clinical Characteristics

As delineated in Table 1, the study embraced 58 patients diagnosed with GBM. The male-to-female ratio was 1.5:1, with a median age [IQR: 25th–75th percentile] of 51.00 [39.00–58.00] years. There were 20 NND patients. The male-to-female ratio was 1:1, with a median age [IQR: 25th–75th percentile] of 26.50 [8.50–35.50] years. The two groups were comparable in gender distribution (χ^2^ = 0.58, *p* = 0.422). However, there was a significant difference in age distribution between the two groups (*p* < 0.001), with the GBM patients group having a higher proportion of participants aged 60 years or older (27.6%) than the NND group (0%).

All cancer patients had the histopathological diagnosis of GBM (grade 4 glioma). Regarding the clinical severity of the GBM group, 32.8% of GBM patients had an ECOG performance status of 1, while 67.2% had an ECOG of 2. Regarding tumor characteristics, 50% of GBM tumors were located in the left hemisphere, 39.7% in the right hemisphere, and 10.3% were in multiple sites. The tumor size was assessed in the GBM group. A total of 22 (37.9%) of the GBM patients had tumors smaller than 5 cm in size, while 36 (62.1%) had tumors 5 cm or larger. In the GBM group, 45 patients (77.6%) had no family history of GBM, whereas 13 patients (22.4%) had a positive family history. Concerning surgical intervention, 62.1% of GBM patients underwent biopsy, 32.6% underwent gross total resection (GTR), and 5.2% underwent subtotal resection (STR). The therapeutic response in the GBM group was evaluated; fifteen (25.9%) GBM patients had CR, eight (13.8%) had PR, seven (12.1%) had SD, and twenty-eight (48.3%) had no response (NR) to therapy.

In the GBM group, the median [IQR] PFS was 6.00 months [4.00, 19.00]. GBM progression was observed in 31 cases (53.4%), whereas it was absent in 27 patients (46.6%). The median [IQR] OS was 10.00 months [6.00–23.00]. Regarding patient status, at the end of the study, thirty-five patients (60.3%) in the GBM group were alive, twenty-one patients (36.2%) had died, and two patients (3.4%) were lost to follow-up.

### 2.4. Distinct DNA Methylation Profiles Between the GBM and NDD Groups

As depicted in Table 1 and Figure 1E, the distribution of promoter methylation patterns across the two groups showed that the NND group had a substantially greater median [IQR] of MGMT promoter methylation (98.00 [97.00, 100.00]) than the GBM group (66.00 [13.00, 97.00]) (*p* < 0.001, Mann–Whitney U test). Similarly, the NND group’s NUPR1 promoter methylation level showed a substantially higher median [IQR] (98.00 [95.00, 100.00]) than the GBM group’s (65.00 [23.00, 87.00]) (*p* < 0.001, Mann–Whitney U test). Additionally, there was a significant difference in NDRG2 promoter methylation pattern between the GBM group (67.00 [54.25, 75.00]) and the NND group (median [IQR]: 19.00 [12.50, 21.50]) (*p* < 0.001, Mann–Whitney U test). Moreover, the GLI1 promoter methylation level was substantially greater in the GBM group (40.50 [26.25, 55.50]) compared to the NND group (median [IQR]: 20.00 [12.75, 22.00]) (*p* < 0.001).

### 2.5. Different Promoter Methylation Patterns of MGMT, NUPR1, NDRG2, and GLI1 Genes Among the Studied Groups

Promoter methylation status was categorized as low or high based on a predetermined threshold for each gene using ROC analysis to detect the best cut-off point that has higher discriminative power. The promoter methylation status of the four genes was compared between NND and GBM samples, as shown in Table 2. The results showed a significant difference in the promoter methylation status of all four genes between the NND and GBM groups. For the MGMT gene, there was a substantial difference in the promoter methylation level between the NND and GBM samples. A higher methylation level was detected in all NND samples, but among the GBM samples, just about 50% (30 out of 58, 51.7%) displayed this high methylation level (χ^2^ = 15, *p* < 0.0001). For other genes, the promoter region of the NUPR1 gene was hypermethylated in 75% of NND samples and 13.8% of GBM samples (χ^2^ = 26.7, *p* < 0.001). The promoter region of the NDRG2 gene was hypermethylated in 5% of NND samples and 96.5% of GBM samples (χ^2^ = 63.3, *p* < 0.001). GLI1 methylation was a distinguishing feature between the NND and GBM samples, with GBM exhibiting a high frequency of GLI1 methylation and the NND samples exhibiting low GLI1 methylation (*p* < 0.001).

### 2.6. Associations Between Clinical Characteristics and Promoter Methylation Patterns of MGMT, NUPR1, NDRG2, and GLI1 Genes in GBM Patients

**Figure 2:**
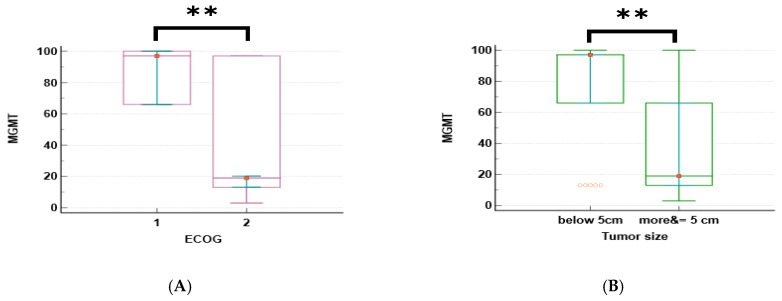

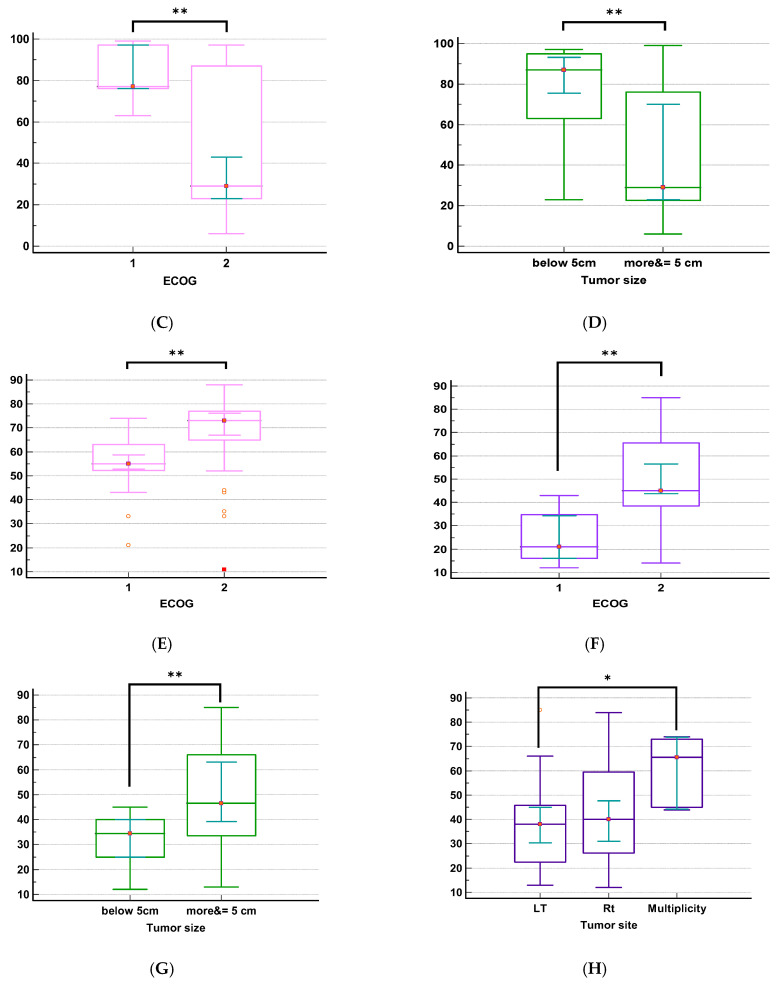
Associations between ECOG status, tumor size, tumor site (*x*-axis), and MGMT, NUPR1, NDRG2, and GLI1 gene promoter methylation patterns (*y*-axis). (**A**) Associations between the promoter methylation pattern of the MGMT gene and ECOG status. (**B**) Associations between the promoter methylation pattern of the MGMT gene and tumor size. (**C**) Associations between the promoter methylation pattern of the NUPR1 gene and ECOG status. (**D**) Associations between the promoter methylation pattern of the NUPR1 gene and tumor size. (**E**) Associations between the promoter methylation pattern of the NDRG2 gene and ECOG status. (**F**) Associations between the promoter methylation pattern of the GLI1 gene and ECOG status. (**G**) Associations between the promoter methylation pattern of the GLI1 gene and tumor size. (**H**) Associations between the promoter methylation pattern of the GLI1 gene and tumor site. Graphs were created using MedCalc^®^ version 22.009 software. Statistical analyses utilized were the Mann–Whitney U test and the Kruskal–Wallis test. The level of significance *p* value was set at 0.05. [MGMT: O6-methylguanine-DNA methyltransferase, NUPR1: Nuclear Protein 1, NDRG2: N-Myc Downstream-Regulated Gene 2, GLI1: glioma-associated oncogene homolog 1, ECOG: Eastern Cooperative Oncology Group, *; *p* value < 0.05, **; *p* value < 0.001].

The promoter methylation patterns of four genes (MGMT, NUPR1, NDRG2, and GLI1) were analyzed in relation to the clinical characteristics of GBM patients. The median and interquartile range (IQR) of methylation for each gene are shown in Table 3. The promoter methylation levels of MGMT and NUPR1 genes were significantly higher in patients with an ECOG status of 1 than in those with an ECOG of 2 (*p* < 0.001 for both genes), as shown in Figure 2A,C. The promoter methylation levels of NDRG2 and GLI1 genes were significantly lower in patients with an ECOG of 1 than in those with an ECOG of 2 (*p* < 0.001 for both genes), as shown in Figure 2E,F. The promoter methylation levels of MGMT and NUPR1 genes were significantly higher in patients with a tumor size of less than 5 cm than in those with a tumor size greater than or equal to 5 cm (*p* = 0.002 and *p* = 0.006, respectively), as shown in Figure 2B,D. The promoter methylation level of GLI1 was significantly lower in patients with a tumor size of less than 5 cm than in those with a tumor size greater than or equal to 5 cm (*p* = 0.005), as shown in Figure 2G. There was no significant difference in the methylation of any gene according to the age, gender, or tumor site of the patients, except for GLI1, which showed a significantly higher promoter methylation in patients with multiple tumor sites than in those with a single tumor site (*p* = 0.045), as shown in Figure 2H.

### 2.7. Correlations Between DNA Methylation Markers and GBM Prognosis, and Therapeutic Outcomes

**Figure 3:**
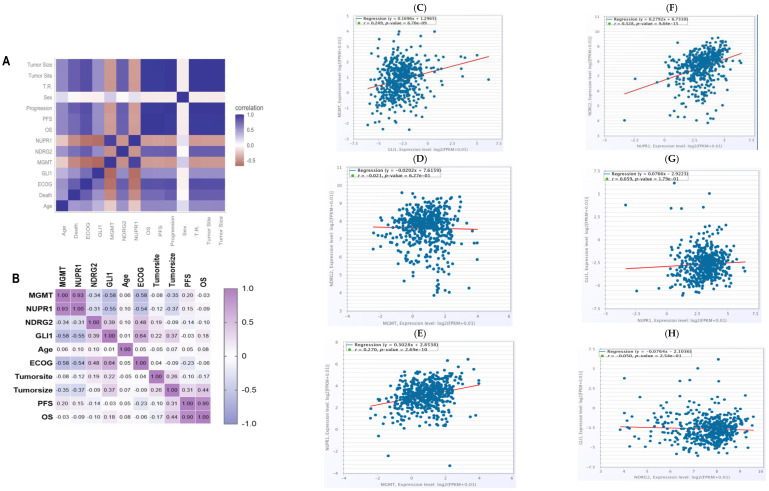
Comprehensive integrated correlation analysis of MGMT, NUPR1, NDRG2, and GLI1 methylation and expression. This composite figure illustrates the multi-layered associations between the four-gene epigenetic signature and clinical/transcriptional outcomes. (**A**,**B**) Correlation matrices depicting the relationship between promoter methylation patterns and clinicopathological characteristics in the GBM discovery cohort (*n* = 58). (**A**) Heatmap visualization of correlation strength; a coefficient of 1.0 (blue) indicates strong positive correlation, while brown indicates strong negative correlation. This panel was generated using R software (v4.3.2), with ggplot2, reshape2, and RColorBrewer packages. (**B**) Numerical correlation matrix providing exact coefficients; purplish-pink indicates strong positive correlation, and blue indicates a negative correlation. This panel was created using GraphPad Prism (v9.5.1). (**C**–**H**) Orthogonal validation via mRNA expression scatter plots (log2 scale) derived from 529 LGG samples (ENCORI Pan-Cancer analysis project: https://rnasysu.com/encori/panGeneCoExp.php), detailing specific gene-to-gene correlations including (**C**) GLI1 vs. MGMT; (**D**) MGMT vs. NDRG2; (**E**) MGMT vs. NUPR1; (**F**) NUPR1 vs. NDRG2; (**G**) GLI1 vs. NUPR1; and (**H**) GLI1 vs. NDRG2. Statistical analyses were performed using Spearman’s correlations. The level of significance *p* value was set at 0.05. [MGMT: O6-methylguanine-DNA methyltransferase, NUPR1: Nuclear Protein 1, NDRG2: N-Myc Downstream-Regulated Gene 2, GLI1: glioma-associated oncogene homolog 1, ECOG: Eastern Cooperative Oncology Group; OS: overall survival, PFS: progression-free survival, T.R.: therapeutic response].

Spearman’s correlations were utilized to analyze the association between the promoter methylation levels of the four genes with one another and the treatment responses, along with prognosis, as shown in Figure 3. The promoter methylation status of MGMT and NUPR1 genes showed a robust positive correlation at a correlation coefficient of r = 0.93; *p* < 0.001. The promoter methylation level of the MGMT gene was negatively correlated with the promoter methylation level of the GLI1 gene at a correlation coefficient of −0.58; *p* < 0.001. The promoter methylation status of the NDRG2 and GLI1 genes exhibited a moderate positive correlation (r = 0.4, *p* < 0.001), while the promoter methylation status of the NUPR1 gene was negatively correlated with the promoter methylation status of the GLI1 gene at a correlation coefficient of −0.55; *p* < 0.001. Promoter hypermethylation of MGMT and NUPR1 genes was associated with better clinical outcomes, with correlation coefficients of −0.613 and −0.577, respectively; *p* < 0.001. Promoter hypermethylation of NDRG2 and GLI1 genes was associated with worse clinical outcomes, with correlation coefficients of 0.463 and 0.448, respectively; *p* < 0.001. ECOG status was negatively correlated with the promoter methylation levels of MGMT (r = −0.58) and NUPR1 (r = −0.54) genes. OS and PFS showed a substantial positive correlation at r = 0.9; *p* < 0.001. The tumor site demonstrated a weak inverse correlation with NUPR1 promoter methylation (r = −0.12) and weak positive correlations with GLI1 (r = 0.22) and NDRG2 (r = 0.19) promoter methylation. For all, the in silico correlations by ENCORI Pan-Cancer Analysis Platform, https://rnasysu.com/encori/panCancer.php, of the genes across the low-grade brain glioma cancer project (Accessed first and revised 25 July 2024) are presented in Figure 3.

### 2.8. Association Between the Methylation Level of MGMT, NUPR1, NDRG2, and GLI1 Genes and Therapeutic Response in GBM Patients (n = 58)

**Figure 4:**
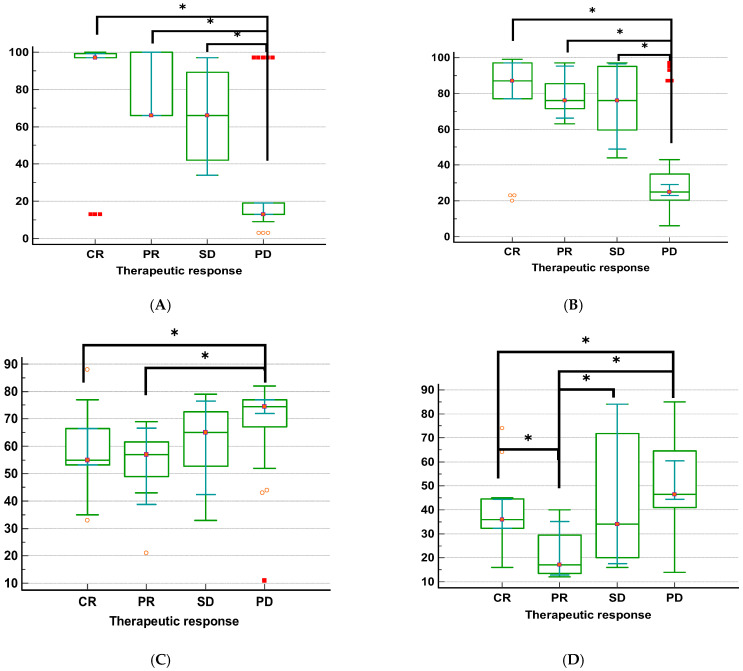
Relationship between treatment response and investigated MGMT, NUPR1, NDRG2, and GLI1 gene promoter methylation: (**A**) different promoter methylation patterns of the MGMT gene across various therapeutic responses; (**B**) different promoter methylation patterns of the NUPR1 gene across various therapeutic responses; (**C**) different promoter methylation patterns of the NDRG2 gene across various therapeutic responses; (**D**) different promoter methylation patterns of the GLI1 gene across various therapeutic responses. Figures were generated using MedCalc^®^ version 22.009 software. The statistical method used was the Kruskal–Wallis H test (KWt) and Conover for post hoc analysis. The level of significance *p* value was set at 0.05. [MGMT: O6-methylguanine-DNA methyltransferase, NUPR1: Nuclear Protein 1, NDRG2: N-Myc Downstream-Regulated Gene 2, GLI1: glioma-associated oncogene homolog 1, CR: complete response, PR: partial response, SD: stable disease, PD: progressed disease, * *p* < 0.05].

The relation between treatment response and the investigated MGMT, NUPR1, NDRG2, and GLI1 gene methylation in GBM patients is shown in Table 4 and Figure 4, according to the Kruskal–Wallis H test (KWt) values. Each of the four methylation markers was strongly associated with therapeutic response. Individuals with complete remission displayed significantly higher promoter methylation levels of the MGMT and NUPR1 genes compared to those with progressive disease (*p* < 0.001, for both genes). Conversely, patients with progressive disease exhibited higher promoter methylation levels of the NDRG2 and GLI1 genes than those who experienced complete remission or partial remission (*p* = 0.004 and *p* = 0.0008, respectively). The highest promoter methylation levels of MGMT and NUPR1 genes were observed in patients who had CR, while the lowest promoter methylation levels of these genes were observed in patients with PD. The highest promoter methylation levels of NDRG2 and GLI1 genes were observed in patients who had PD, while the lowest promoter methylation level of the NDRG2 gene was observed in patients who had CR, and the lowest promoter methylation level of the GLI1 gene was detected in patients with PR.

### 2.9. The Impact of MGMT, NUPR1, NDRG2, and GLI1 Gene Promoter Methylation on PFS in GBM Patients (n = 58)

**Figure 5:**
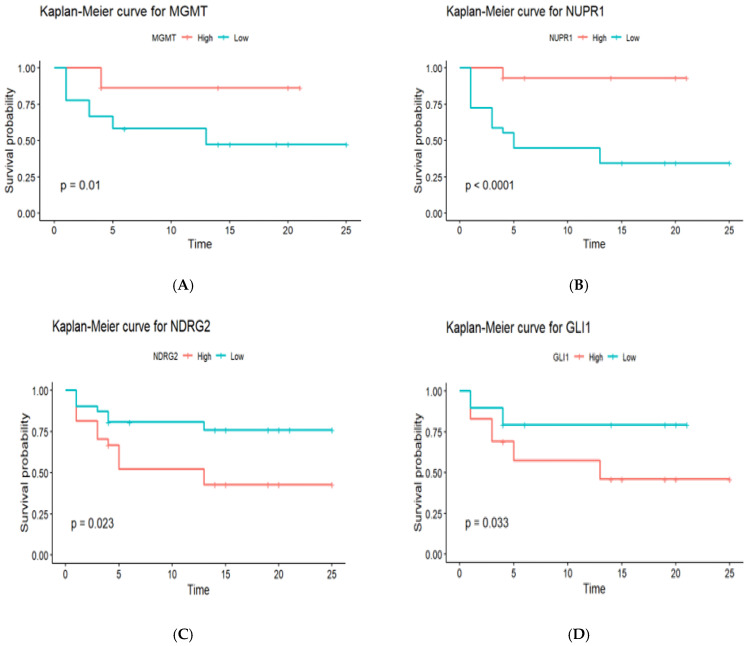
Impact of promoter methylation status of MGMT, NUPR1, NDRG2, and GLI1 genes on PFS in GBM patients (*n* = 58). (**A**) Kaplan–Meier curve for PFS of different methylation levels of the MGMT gene [high vs. low]. (**B**) Kaplan-Meier curve for PFS of different methylation levels of the NUPR1 gene [high vs. low]. (**C**) Kaplan–Meier curve for PFS of different methylation levels of the NDRG2 gene [high vs. low]. (**D**) Kaplan–Meier curve for PFS of different methylation levels of the GLI1 gene [high vs. low]. The Kaplan–Meier curves were generated using R software (version 4.3.2), and the following packages, survival, survminer, and ggplot2, were utilized to create graphs. The level of significance *p* value was set at 0.05. [GBM: glioblastoma, MGMT: O6-methylguanine-DNA methyltransferase, NUPR1: Nuclear Protein 1, NDRG2: N-Myc Downstream-Regulated Gene 2, GLI1: glioma-associated oncogene homolog 1].

To ascertain how gene promoter methylation affected the PFS of GBM patients, Kaplan–Meier survival analysis curves were generated, as shown in Figure 5. The survival curves of hypomethylated and hypermethylated genes were compared using the log-rank test. Promoter hypermethylation of the MGMT gene was associated with significantly better PFS compared to promoter hypomethylation (18.5 months vs. 12.3 months, *p* = 0.01). Similar to MGMT, promoter hypermethylation of the NUPR1 gene showed significantly better PFS compared to promoter hypomethylation (21.2 months vs. 9.8 months, *p* < 0.0001). Promoter hypomethylation of the NDRG2 gene was associated with better PFS compared to promoter hypermethylation (19.4 months vs. 11.6 months, *p* = 0.023). Similar to NDRG2, promoter hypomethylation of the GLI1 gene was associated with better PFS compared to promoter hypermethylation (16.7 months vs. 13.9 months, *p* = 0.033).

### 2.10. The Predictive Value of MGMT, NUPR1, NDRG2, and GLI1 Gene Promoter Methylation on OS of GBM Patients (n = 58)

**Figure 6:**
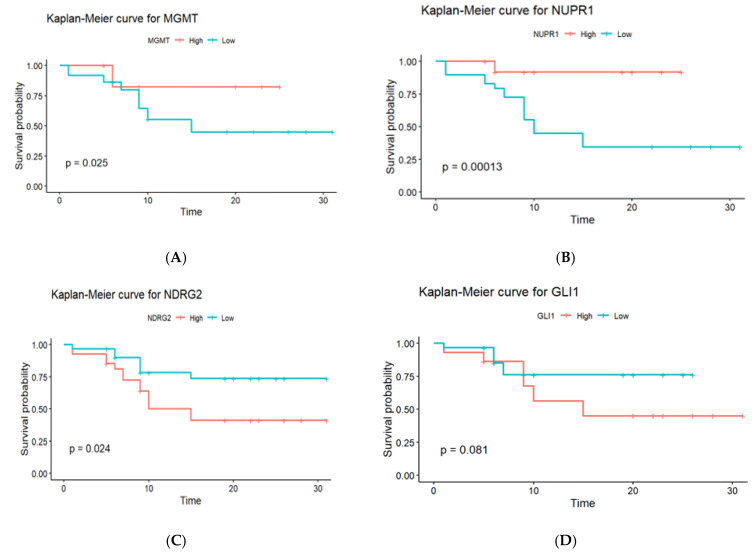

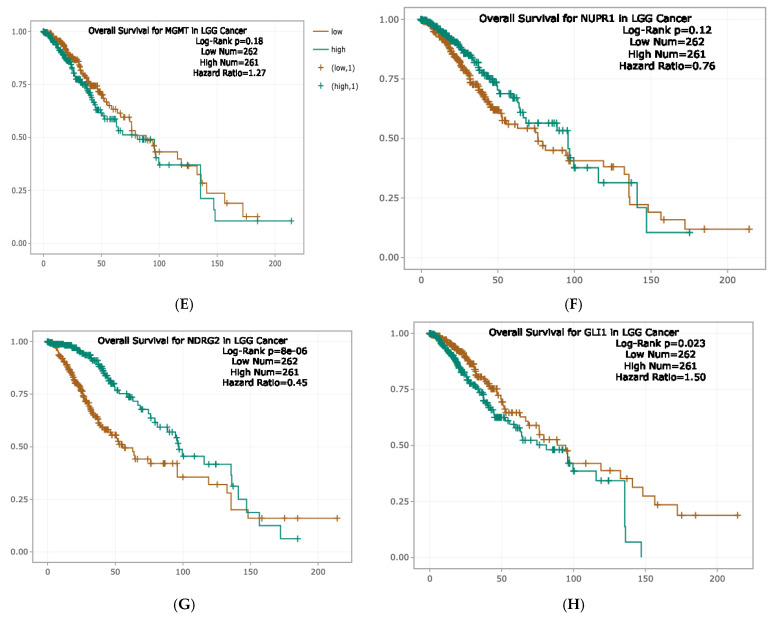
Kaplan–Meier curves for OS stratified by gene methylation in GBM. (**A**) Kaplan–Meier curve for OS of different methylation levels of the MGMT gene [high vs. low], (**B**) Kaplan–Meier curve for OS of different methylation levels of the NUPR1 gene [high vs. low]. (**C**) Kaplan–Meier curve for OS of different methylation levels of the NDRG2 gene [high vs. low]. (**D**) Kaplan–Meier curve for OS of different methylation levels of the GLI1 gene [high vs. low]. (**E**) KM curve for survival of MGMT in LGG. (**F**) KM curve for survival of NUPR1 in LGG. (**G**) KM curve for survival of NDRG2 in LGG. (**H**) KM curve for survival of GLI1 in LGG, retrieved from ENCORI, https://rnasysu.com/encori/panGeneSurvivalExp.php#LGG; data scale is log2 scale. The Kaplan–Meier curves (**A**–**D**) were generated using R software (version 4.3.2), and the following packages, survival, survminer, and ggplot2, were utilized to create graphs. The level of significance *p* value was set at 0.05, with a *p* value for NUPR1 of 0.12, 0,18 for MGMT, 8 × 10^−6^ for NDRG2, and 0.023 for GLI1. [GBM: glioblastoma, LGG: Lower Grade Glioma, KM: Kaplan–Meier, MGMT: O6-methylguanine-DNA methyltransferase, NUPR1: Nuclear Protein 1, NDRG2: N-Myc Downstream-Regulated Gene 2, GLI1: glioma-associated oncogene homolog 1].

Kaplan–Meier survival analysis curves were constructed to determine the effects of gene promoter methylation on the OS of GBM patients. The log-rank test was used to compare the survival curves between patients with hypomethylated and hypermethylated genes. Three of the four genes studied exhibited statistically significant variations in OS, depending on methylation status, as shown in Figure 6. Promoter hypermethylation of the MGMT gene was associated with a better prognosis in GBM patients, as seen by a longer median (OS). Patients who had elevated MGMT methylation had a median OS of 18.5 months, which was considerably longer than the 12.3 months observed in patients with low methylation levels (*p* = 0.025). The Kaplan–Meier survival curves clearly distinguish between these two groups, demonstrating the prognostic usefulness of MGMT methylation for patient outcomes. Similar to the MGMT gene, promoter hypermethylation of the NUPR1 gene was associated with a better prognosis. Patients with high NUPR1 promoter methylation had a longer median OS of 21.2 months compared to 9.8 months in patients with low NUPR1 promoter methylation, with a statistically significant difference (*p* = 0.00013). In contrast, promoter hypermethylation of the NDRG2 gene was associated with a poorer prognosis. Patients with high NDRG2 promoter methylation had a median OS of 11.6 months, which was significantly shorter than the 19.4-month median OS for patients with low NDRG2 promoter methylation (*p* = 0.024). Regarding the GLI1 gene’s methylation, there was no statistically significant variation in OS. Patients with high GLI1 promoter methylation had a somewhat reduced median overall survival (OS) of 13.9 months compared to 16.7 months for those with low promoter methylation. However, the difference (*p* = 0.081) did not reach statistical significance.

#### Gene Promoter Methylation and Clinicopathological Characteristics as Predictors of OS in GBM Patients: A Univariate and Multivariate Cox Regression Analysis

A Cox proportional hazard regression model was used to assess the effects of various factors on OS. Among these factors are the promoter methylation status of genes, age (<60 vs. ≥60 years), sex (male vs. female), ECOG scores (ECOG 2 or less), tumor site (right vs. left vs. multiple), tumor size (<5 cm vs. ≥5 cm), and surgical resection status (biopsy, total gross resection (TGR) vs. partial resection (PR)), as shown in Table 5. In the univariate analysis, the multiple tumor site was significantly associated with a higher risk of mortality (HR = 4.44, 95% CI = 1.42–13.88, *p* = 0.010), whereas NUPR1 promoter hypermethylation was significantly associated with a lower risk of mortality (HR = 0.97, 95% confidence interval [CI] = 0.96–0.99, *p* = 0.002). A significant association was also found between surgical intervention in patients who underwent STR and OS (HR for STR = 12.12, 95% CI = 2.72–53.93, *p* = 0.001). However, no significant associations were detected regarding the promoter methylation status of NDRG2 and GLI1 genes, age, tumor size, and gender. In multivariable analysis, the promoter hypermethylation of the NUPR1 gene remained significantly linked with a lower risk of mortality (HR = 0.91, 95% CI = 0.87–0.95, *p* < 0.001). However, tumor size became strongly associated with mortality (HR for ≥5 cm = 9.81, 95% CI = (2.4 to 39.7), *p* = 0.0014). Surgical intervention was still substantially linked with survival (HR for STR = 223.35, 95% CI = 12.70–3927.93, *p* < 0.001). The multivariate analysis revealed a significant association between gender and OS; female gender was significantly associated with improved OS (HR for female = 0.09, 95% CI = 0.01–0.72, *p* = 0.024). There were no significant associations detected with promoter methylation levels of NDRG2 and GLI1 genes, age, or tumor site.

A stepwise selection procedure was performed to identify the significant variables for predicting OS (Table 6). The covariates included in the model were the promoter methylation levels of the NUPR1, NDRG2, and GLI1 genes, as well as age and tumor size. The C-index for the Cox regression model was 0.729, indicating that the model was able to predict the risk of mortality with moderate accuracy. The results revealed that the promoter hypermethylation of the NUPR1 gene was associated with an increase in OS (*p* = 0.0003). A one-unit increase in the NUPR1 promoter methylation level was linked to a 0.9564-fold decrease in mortality risk, implying that patients with NUPR1 promoter hypermethylation have a longer median survival time. Neither NDRG2 nor GLI1 promoter methylation was linked to OS (*p* = 0.8361 and 0.9886, respectively). No statistically significant correlation between age at diagnosis and OS (*p* = 0.7620). Regarding tumor size, a larger tumor size was substantially linked to a shorter OS rate (*p* = 0.0014). Tumor burden has a significant effect on survival; a one-unit increase in tumor size was linked to a 9.8143-fold increase in the risk of death. Consequently, tumor size and NUPR1 promoter methylation were found to be independent predictors of GBM patients’ overall survival (OS).

### 2.11. The Role of Promoter Methylation of NUPR1, NDRG2, and GLI1 Genes to Distinguish Between NND and GBM Groups

**Figure 7:**
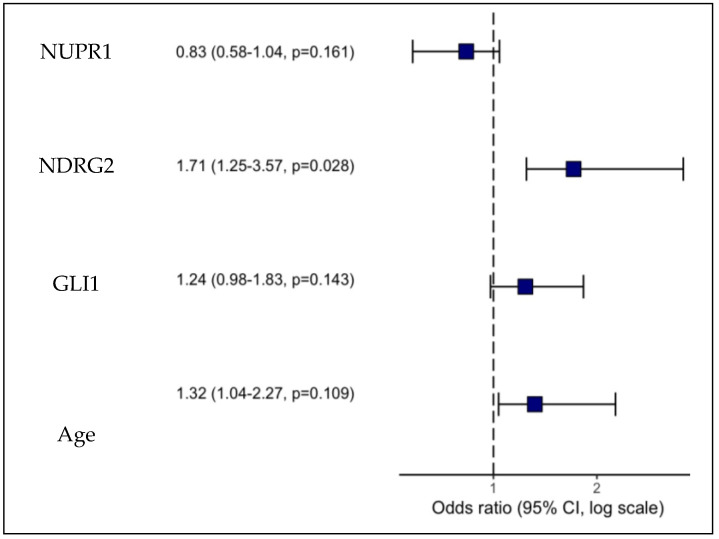
Multivariate binary logistic regression analysis forest plot of NUPR1, NDRG2, and GLI1 genes, as well as the age. The NDRG2 gene promoter hypermethylation is an independent predictor for the development of GBM (*n =* 58). The forest plot was generated using R software (version 4.3.2), and the following packages, dplyr, stat, ggplot2, and broom, were utilized. The statistical methods used were univariate and multivariate binary logistic regression analysis. The level of significance *p* value was set at 0.05. [NUPR1: Nuclear Protein 1, NDRG2: N-Myc Downstream-Regulated Gene 2, GLI1: glioma-associated oncogene homolog 1, CI: confidence interval].

Binary logistic regression analysis was performed to assess the association between the promoter methylation status of four investigated genes and GBM disease, as indicated by the odds ratios (ORs) and corresponding (CI) univariate logistic regression analysis, which demonstrated significant associations between promoter methylation of NDRG2 and GLI1 and the development of GBM. Promoter hypermethylation of the NDRG2 gene increased the OR for GBM development by 1.45 (1.25–1.85, *p* < 0.001), while promoter hypermethylation of the GLI1 gene increased the OR by 1.26 (1.14–1.45, *p* < 0.001). The analysis also revealed the involvement of the aberrant promoter methylation of the NUPR1 gene in GBM evolution. Promoter hypermethylation of the NUPR1 gene decreased the odds ratio for GBM development by 0.83 (0.74–0.91, *p* < 0.001), indicating a potential protective effect. The analysis also revealed that age is a significant factor in GBM evolution, with each additional year of age associated with a 1.23-fold increase in GBM risk (95% CI: 1.12–1.38, *p* < 0.001). The correlation between MGMT and NUPR1 was high. A stepwise selection procedure was performed to identify significant variables in predicting GBM disease. Variables included in the multivariate analysis were promoter methylation levels of the NUPR1, NDRG2, and GLI1 genes, as well as age. Univariate logistic regression analysis identified promoter methylation levels of the NUPR1, NDRG2, GLI1 genes, as well as age, as significant predictors of GBM disease (*p* < 0.05), as shown in Table 7, However, in multivariate analysis adjusted for covariates, only promoter methylation of the NDRG2 gene remained a significant independent predictor of GBM disease (OR = 1.71, 95% CI (1.25–3.57), *p* = 0.028), as presented in Figure 7 and Table 8. The final multivariate logistic regression model, including promoter methylation of NDRG2, GLI1, NUPR1, and age, had excellent discriminative ability, as indicated by an AUC of 0.997. Goodness of fit testing using the Hosmer–Lemeshow test was non-significant (chi-sq (8) 0.27, *p* = 1.000), confirming that the model fit the data well.

### 2.12. Promoter Methylation Patterns of NUPR1, MGMT, NDRG2, and GL1 Genes: An ROC Analysis of Their Discriminative, Prognostic, and Predictive Potential

**Figure 8:**
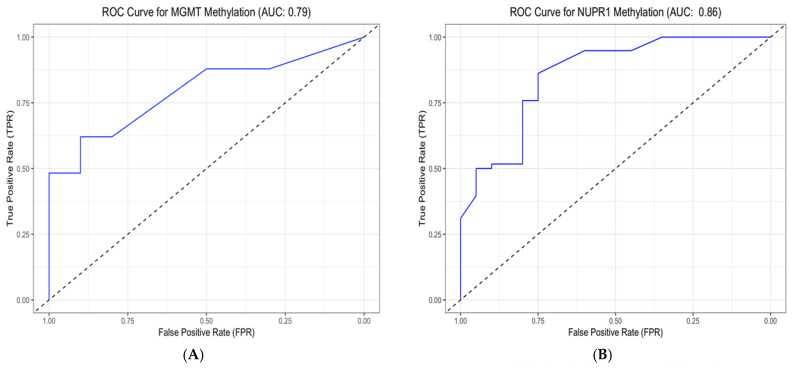

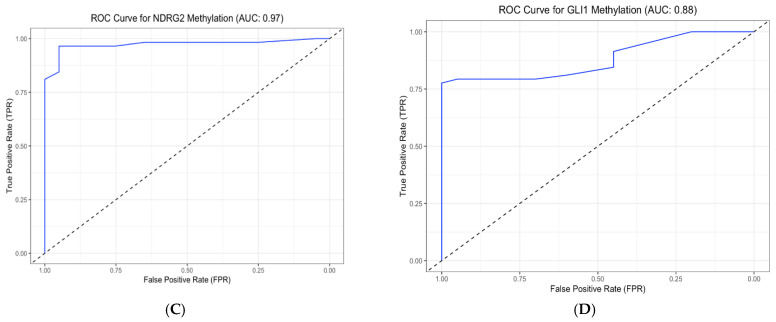
ROC curves for the MGMT, NUPR1, NDRG2, and GLI1 gene methylation (**A**–**D**) as epigenetic biomarkers to differentiate between neoplastic (GBM) and non-neoplastic (NND) brain diseases. The ROC curves were generated using R software (version 4.3.2), and the following packages, pROC, ROCR, PRROC, and plotROC, were utilized. The level of significance *p* value was set at 0.05. [AUC: area under the curve, MGMT: O6-methylguanine-DNA methyltransferase, NUPR1: Nuclear Protein 1, NDRG2: N-Myc Downstream-Regulated Gene 2, GLI1: glioma-associated oncogene homolog 1].

**Figure 9:**
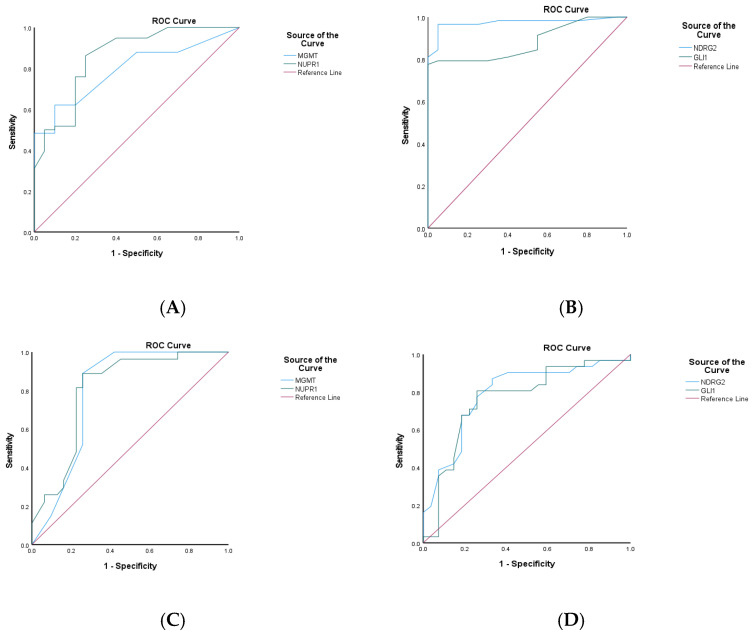

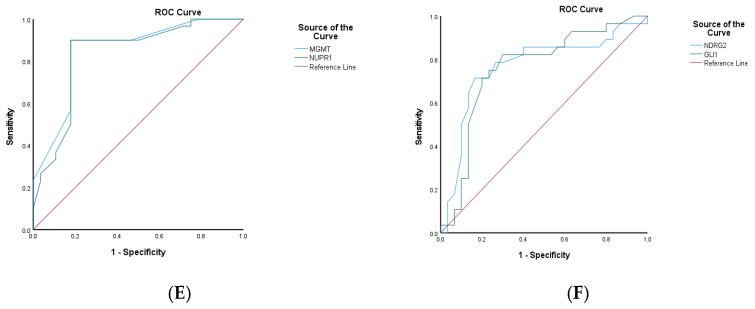
Comparison of model performance using ROC analysis. (**A**) represents the discriminative power of the MGMT and NUPR1 genes, and (**B**) represents the discriminative power of the NDRG2 and GLI1 methylation markers. (**C**) The prognostic power of MGMT and NUPR1 genes. (**D**) The prognostic ability of the NDRG2 and GLI1 methylation markers. (**E**) Predictive power of the MGMT and NUPR1 genes and (**F**) predictive power of the NDRG2 and GLI1 methylation markers. The ROC curves were generated using SPSS software (version 27.0). The level of significance *p* value was set at 0.05. [MGMT: O6-methylguanine-DNA methyltransferase, NUPR1: Nuclear Protein 1, NDRG2: N-Myc Downstream-Regulated Gene 2, GLI1: glioma-associated oncogene homolog 1].

**Figure 10:**
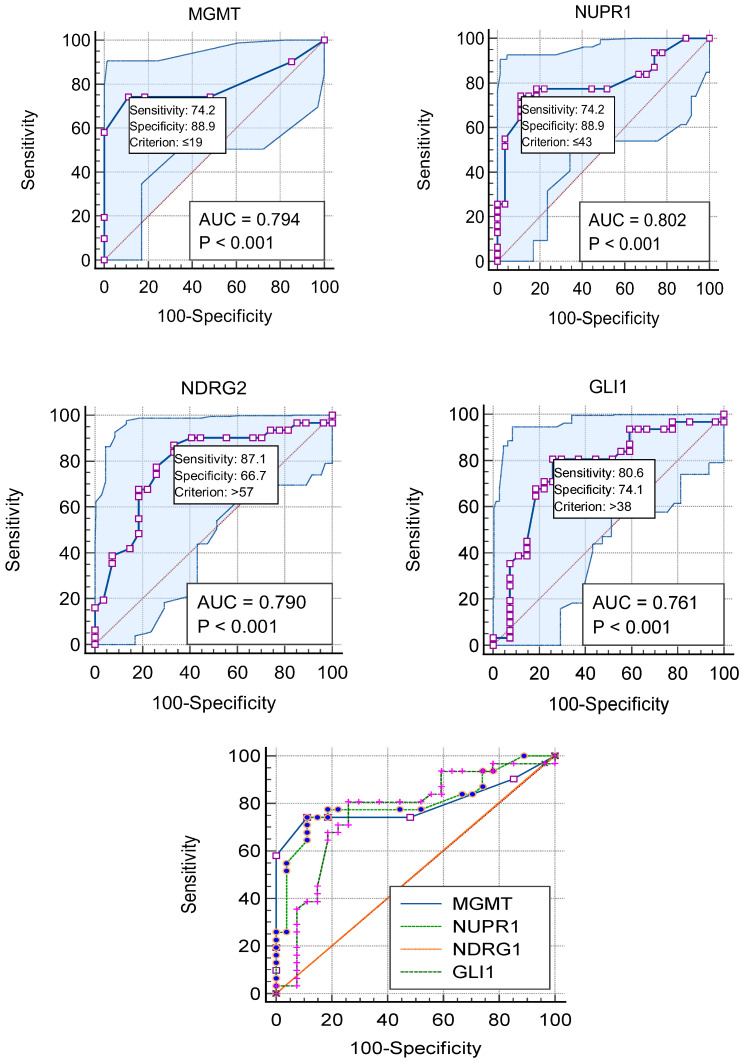
Receiver operating characteristic (ROC) curves for the MGMT, NUPR1, NDRG2, and GLI1 gene methylation as methylation markers for predicting the prognosis of GBM patients. The ROC curves were generated using MedCalc^®^ version 22.009 software. The level of significance *p* value was set at 0.05. [MGMT: O6-methylguanine-DNA methyltransferase, NUPR1: Nuclear Protein 1, NDRG2: N-Myc Downstream-Regulated Gene 2, GLI1: glioma-associated oncogene homolog 1].

The discriminative, prognostic, and predictive potential of four gene methylation markers was assessed using an AUC in ROC analysis. The results are presented in Figure 8, Figure 9 and Figure 10 and Table 9, including the AUC, standard error (SE), significance, and the corresponding 95% CI. The discriminative potential power of investigated methylation biomarkers to differentiate between malignant and non-malignant conditions was assessed using ROC analysis, as depicted in Figure 8 and Figure 9A,B. The results showed that the promoter methylation level of the NUPR1 gene demonstrated a higher AUC of 0.858 (S.E. = 0.050, *p* < 0.001), indicating its discriminative ability in distinguishing between GBM and NND diseases. Similarly, promoter methylation of the MGMT gene exhibited a significant AUC of 0.793 (S.E. = 0.051, *p* < 0.001), suggesting its discriminative potential. Promoter methylation of the NDRG2 and GL1 gene also showed excellent discriminative ability, with high AUC values of 0.973 (S.E. = 0.018, *p* < 0.001) and 0.881 (S.E. = 0.037, *p* < 0.001), respectively.

The prognostic potential of the investigated methylation markers in predicting clinical outcomes for GBM patients was evaluated using ROC analysis, as shown in panels C and D in Figure 9 and Figure 10. Regarding the overall prognostic ability, promoter methylation of the NUPR1 gene displayed a high AUC of 0.802 (S.E. = 0.060, *p* < 0.001), indicating its ability to predict the prognosis of the GBM patients. Similarly, MGMT exhibited a comparable AUC of 0.794 (S.E. = 0.064, *p* < 0.001). Promoter methylation of the NDRG2 and GL1 genes also showed significant prognostic ability, with AUC values of 0.790 (S.E. = 0.062, *p* < 0.001) and 0.761 (S.E. = 0.066, *p* = 0.001), respectively.

In evaluating the ability to predict therapeutic response, as shown in Figure 9E,F, the promoter methylation of the NUPR1 gene showed a robust AUC of 0.832 (S.E. = 0.057, *p* < 0.001). This high AUC value indicates a strong predictive capability, suggesting that NUPR1 promoter methylation could serve as a reliable biomarker for assessing how patients with GBM might respond to a standard therapeutic regimen. The statistical significance (*p* < 0.001) further reinforces the potential clinical utility of this marker in guiding therapeutic decisions and improving patient outcomes. Similarly, promoter methylation of the MGMT gene exhibited a high AUC of 0.848 (S.E. = 0.053, *p* < 0.001), indicating its strong predictive potential. Promoter methylation of the NDRG2 and GL1 genes also showed significant predictive ability, with AUC values of 0.767 (S.E. = 0.067, *p* < 0.001) and 0.758 (S.E. = 0.067, *p* = 0.001), respectively.

The promoter methylation status of the MGMT and NUPR1 genes has been shown to negatively correlate with GBM disease. According to ROC analysis in Figure 8 and Figure 9A,B, the optimal cut-off point of the methylated MGMT gene was 77.5 (sensitivity: 0.621; specificity: 0.900), and the test was considered positive if its value was less than or equal to 77.5, while the optimal cut-off point of the methylated NUPR1 gene was 96.5 (sensitivity: 0.862; specificity: 0.750). Promoter methylation status of the NDR2 and GLI1 genes has been shown to positively correlate with the evolution of GBM. The optimal cut-off point of the methylated NDRG2 gene was 32.5 (sensitivity: 96.6%; specificity: 95%), while the optimal cut-off point of the methylated GLI1 gene was 24 (sensitivity: 77.6%; specificity: 100%).

In terms of predictive accuracy of methylation markers to predict therapeutic response, as shown in E and F in Figure 9, the optimal cut-off point of the methylated MGMT gene was 39 (sensitivity: 85.7%; specificity: 62.2%), while the optimal cut-off point of the methylated NUPR1 gene was 68.5 (sensitivity:66.7%; specificity: 67.6%). The optimal cut-off point of the methylated NDRG2 gene was 68.5 (sensitivity:66.7%; specificity: 67.6%), while the optimal cut-off point of the methylated GLI1 gene was 39 (sensitivity: 85.7%; specificity: 62.2%).

### 2.13. Optimal Cut-Off Values of NUPR1, MGMT, GLI1, and NDRG2 Methylation for Predicting GBM Outcomes

Plotting the ROC curves for representative cut-off values of the percentage of promoter methylation levels of MGMT, NUPR1, NDRG2, and GLI1 genes is shown in Figure 10. Based on the highest LR+, sensitivity, specificity, and accuracy, the best cut-off points were chosen. The cut-off values of 19%,43%, 57%, and 38% in this dataset yielded the best results, with the highest LR+, accuracy, and Youden index.

The overall sensitivities, specificities, and other evaluation metrics for the four methylation markers (MGMT, NUPR1, NDRG2, and GLI1) in GBM patients are shown in Table 10. The optimal criteria for determining the positivity of the markers were ≤19 for MGMT, ≤43 for NUPR1, >57 for NDRG2, and >38 for GLI1. AUCs were above 0.75 for all markers, indicating good discriminatory ability. All four markers had statistically significant AUCs (*p* < 0.0001). MGMT and NUPR1 showed the highest AUCs of 0.794 and 0.802, respectively. Sensitivities ranged from 74.19% to 87.10%, with NDRG2 having the highest sensitivity of 87.10%. Specificities were higher, ranging from 66.67% to 88.89% for NDRG2 and MGMT/NUPR1, respectively. Positive likelihood ratios above 3 and negative likelihood ratios below 0.3 indicate good prognostic accuracy for all markers. PPVs were above 75%, and NPVs were above 75% for all markers as well. The Youden index, which measures the overall diagnostic effectiveness of a marker [38], was highest for NUPR1 and MGMT (0.6308), followed by GLI1 (0.5472) and NDRG2 (0.5376).

### 2.14. In Silico Functional Enrichment Analysis

**Figure 11:**
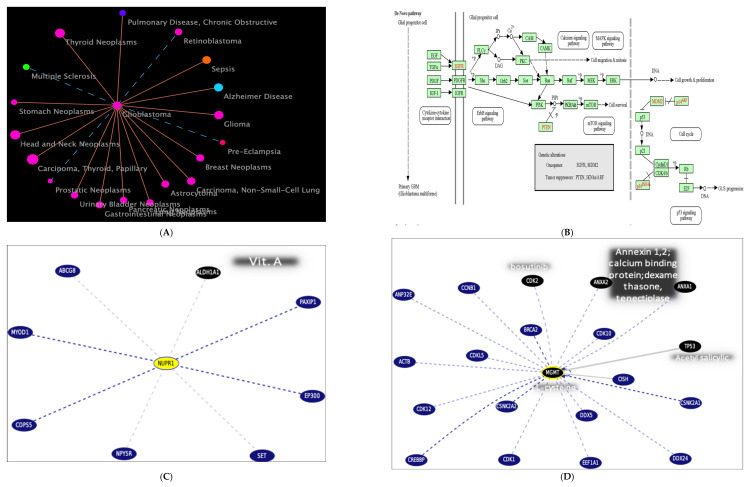

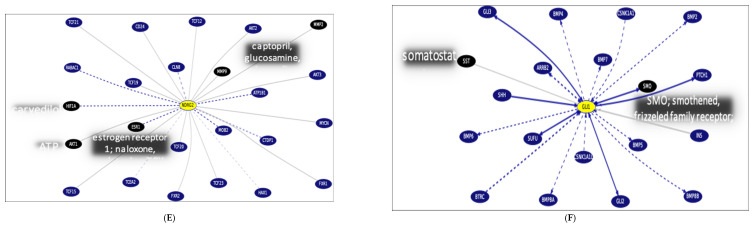

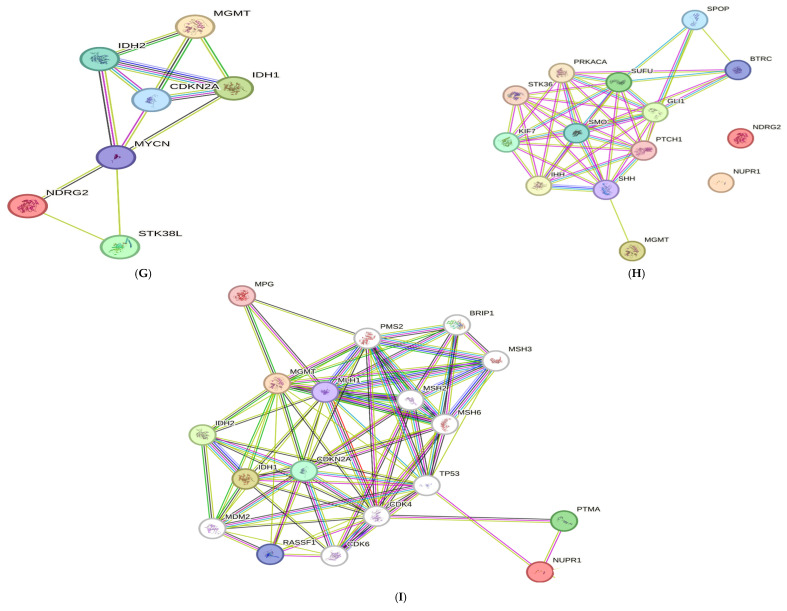
In silico functional enrichment analysis of the methylated genes. (**A**) GBM disease network, http://www.cuilab.cn/hmdd. Disease similarity with a positive edge is presented with an orange line; four negative edge diseases are shown in blue in a complete line. (**B**) Glioma/GBM genetic alteration pathways from KEGG (accessed on 17 October 2023). [GBM: glioblastoma, EGFR: Epidermal Growth Factor Receptor, PDGF: Platelet-Derived Growth Factor, PDGFR: Platelet-Derived Growth Factor Receptor, PI3K: Phosphoinositide 3-Kinase, AKT: Protein Kinase B, mTOR: Mechanistic Target of Rapamycin, PTEN: phosphatase and tensin homolog, MDM2: Mouse Double Minute 2 homolog, p53: Tumor Protein p53, CDK4/6: Cyclin-Dependent Kinase 4/6]. (**C**) https://genome.ucsc.edu/cgi-bin/hgGeneGraph?gene=NUPR1&supportLevel=text&hideIndirect=on&geneCount=25&geneAnnot=drugbank&1=OK&geneCount=25 (accessed on 17 October 2023) [GBM: glioblastoma multiforme, NUPR1: Nuclear Protein 1, ABCG8: ATP Binding Cassette Subfamily G Member 8, MYOD1: Myogenic Differentiation 1, COPS5: COP9 Signalosome Subunit 5, NPY5R: Neuropeptide Y Receptor Y5, SET: SET Nuclear Proto-Oncogene, EP300: E1A Binding Protein P300, PAXIP1: PAX Interacting Protein 1, ALDH1A1: Aldehyde Dehydrogenase 1 Family Member A1]. (**D**) https://genome.ucsc.edu/cgi-bin/hgGeneGraph?gene=MGMT&1=OK&supportLevel=text&hideIndirect=on&geneCount=20&geneAnnot=drugbank&1=OK&geneCount=20 [MGMT: O-6-methylguanine-DNA methyltransferase, CCNB1: Cyclin B1, ANP32E; acidic nuclear phosphoprotein 32 family member E, ACTB: Actin Beta, CDK5: Cyclin-Dependent Kinase 5, CDK2: Cyclin-Dependent Kinase 2, BRCA2: BRCA2 DNA Repair Associated, CISH: cytokine inducible SH2 containing protein, CSKN2A2: casein kinase 2 alpha 2, CDK12: Cyclin-Dependent Kinase 12, CREBBP: CREB Binding Protein, CDK1: Cyclin-Dependent Kinase 1, CSNK2A1: casein kinase 2 alpha 1, DDDX5: DEAD-Box Helicase 5, ANXA2: Annexin A2, CDK10: Cyclin-Dependent Kinase 10, TP53: Tumor Protein P53, DDDX24: DEAD-Box Helicase 24, ANXA1: Annexin A1, EEF1A1: Eukaryotic Translation Elongation Factor 1 Alpha 1]. (**E**) https://genome.ucsc.edu/cgi-bin/hgGeneGraph?gene=NDRG2&1=OK&supportLevel=text&hideIndirect=on&geneCount=25&geneAnnot=drugbank&1=OK&geneCount=25 [NDRG2: N-Myc Downstream-Regulated 2, RAB4C1: RAB4C, Member RAS Oncogene Family, TCF19: Transcription Factor 19, ATP1B1: ATPase Na+/K+ Transporting Subunit Beta 1, HIF1A: Hypoxia Inducible Factor 1 Subunit Alpha, ESR1: Estrogen Receptor 1, MOB2: MOB Kinase Activator 2, CLN8: Ceroid Lipofuscinosis Neuronal 8, CD24: CD24 Molecule, TCF12: Transcription Factor 12, AKT2: AKT Serine/Threonine Kinase 2, MMP2: Matrix Metallopeptidase 2, MMP9: Matrix Metallopeptidase 9, AKT3: AKT Serine/Threonine Kinase 3, MYCN: MYCN Proto-Oncogene, BHLH Transcription Factor, CTDP1: CTD Phosphatase Subunit 1, TCF20: Transcription Factor 20, TCF23: Transcription Factor 23, FXR2: FMR1 Autosomal Homolog 2, HAX1: HCLS1 Associated Protein X 1, TCF15: Transcription Factor 15, TCEA2: Transcription Elongation Factor A2, FXR2: FMR1 Autosomal Homolog 2, FXR1: Fragile X Mental Retardation 1]. (**F**) https://genome.ucsc.edu/cgi-bin/hgGeneGraph?gene=GLI1&1=OK&supportLevel=text&hideIndirect=on&geneCount=20&geneAnnot=drugbank&geneCount=25&1=OK [GLI1: glioma-associated oncogene homolog 1, GLI3: glioma-associated oncogene homolog 3, BMP4: bone morphogenetic protein 4, CSNK1A1: casein kinase 1 alpha 1, BMP2: bone morphogenetic protein 2, BMP7: bone morphogenetic protein 7, SMO: Smoothened, Frizzled Family Receptor, PTCH1: Patched 1, SST: Somatostatin, SHH; Sonic Hedgehog, ARRB2: Beta-Arrestin-2, SUFU; suppressor of fused homolog, BMP5; bone morphogenetic protein 5, INS: insulin, BMP6; bone morphogenetic protein 6, BTRC: beta-transducin repeat-containing protein, BMP8A: bone morphogenetic protein 8a, GLI2: glioma-associated oncogene homolog 2, CSNK1A1L: casein kinase 1 alpha 1 like, BMP8B: bone morphogenetic protein 8b]. (**G**–**I**) SRTING PPI prediction; (**G**) [MGMT: O-6-methylguanine-DNA methyltransferase, IDH2: Isocitrate Dehydrogenase 2, IDH1: Isocitrate Dehydrogenase 1, CDKN2A: Cyclin-Dependent Kinase Inhibitor 2A, MYCN: MYCN Proto-Oncogene, BHLH Transcription Factor, NDRG2: N-Myc Downstream-Regulated 2, STK38L: Serine/Threonine Kinase 38 Like]; (**H**) [SPOP: Speckle Type POZ Protein, BTRC: Beta-Transducin Repeat Containing E3 Ubiquitin Protein Ligase, SUFU: Suppressor Of Fused Homolog (Drosophila), GLI1: glioma-associated oncogene homolog 1, NDRG2: N-Myc Downstream-Regulated 2, NUPR1: Nuclear Protein 1, PRKACA: Protein Kinase cAMP-Activated Catalytic Subunit Alpha, STK36: Serine/Threonine Kinase 36, SMO: Smoothened, Frizzled Class Receptor, PTCH1: Patched 1, KIF7: Kinesin Family Member 7, IHH: Indian Hedgehog, SHH: Sonic Hedgehog, MGMT: O-6-methylguanine-DNA methyltransferase]; (**I**) [MPG: Membrane-Bound O-Acyltransferase Domain Containing 2, PMS2: Postmeiotic Segregation Increased 2 (*S. cerevisiae*), BRIP1: BRCA1 Interacting Protein C-Terminal Helicase 1, MSH3: MutS Homolog 3, MGMT: O-6-methylguanine-DNA methyltransferase, MLH1: MutL Homolog 1, MSH2: MutS Homolog 2, MSH6: MutS Homolog 6, TP53: Tumor Protein P53, PTMA: prothymosin, alpha, NUPR1: Nuclear Protein 1, IDH2: Isocitrate Dehydrogenase (NADP(+)) 2, IDH1: Isocitrate Dehydrogenase (NADP(+)) 1, CDKN2A: Cyclin-Dependent Kinase Inhibitor 2A, MDM2: MDM2 Proto-Oncogene, RASSF1: Ras Association Domain Family Member 1, CDK6: Cyclin-Dependent Kinase 6, CDK4: Cyclin-Dependent Kinase 4].

1. GBM disease similarity (accessed on 15 March 2023) (Figure 11A) is shown at https://www.cuilab.cn/hmdd (accessed on 15 March 2023);
2. The KEGG-targeted pathway (Figure 11B) and glioma/GBM genetic alteration pathways from the KEGG were used (accessed on 17 October 2023);
3. Top gene targets of the tested gene interactions and pathways from curated databases and text mining were used (accessed on July 2024) (Figure 11C–F).

The top gene targets interacting with the NUPR1 gene, as shown in Figure 11C, are ATP Binding Cassette Subfamily G Member 8 (ABCG8), Myogenic Differentiation 1 (MYOD1), COP9 Signalosome Subunit 5 (COPS5), Neuropeptide Y Receptor Y5 (NPY5R), SET Nuclear Proto-Oncogene (SET), E1A Binding Protein P300 (EP300), PAX Interacting Protein 1 (PAXIP1), and Aldehyde Dehydrogenase 1 Family Member A1 (ALDH1A1).

The top genes interacting with the MGMT gene, as shown in Figure 11D, are Tumor Protein P53 (TP53), Cyclin-Dependent Kinase 1 (CDK1), Breast Cancer Type 2 Susceptibility Protein (BRCA2), Actin Beta (ACTB), and COP9 Signalosome Subunit 5 (COPS5). These interactions are crucial for understanding the role of MGMT in DNA repair and its implications in cancer biology.

The top genes interacting with the NDRG2 gene, as shown in Figure 11E, are Estrogen Receptor 1 (ESR1), Hypoxia Inducible Factor 1 Alpha (HIF1A), C-terminal Binding Protein 1 (CTBP1), Histone Deacetylase 4 (HDAC4), Transforming Growth Factor Beta 2 (TGFB2), and Transforming Growth Factor Beta 3 (TGFB3). These interactions are crucial for understanding the role of NDRG2 in various biological pathways and its potential implications in disease mechanisms.

The top genes interacting with the GLI1 gene, as shown in Figure 11F, are Sonic Hedgehog (SHH), Smoothened, Frizzled Family Receptor (SMO), Suppressor of Fused Homolog (SUFU), GLI Family Zinc Finger 2 (GLI2), Bone Morphogenetic Protein 4 (BMP4), Bone Morphogenetic Protein 7 (BMP7), SKI-like Oncogene (SKIL), and Beta-Arrestin 2 (ARRB2). These interactions are crucial for understanding the role of GLI1 in various biological pathways and its potential implications in disease mechanisms.

4. Functional enrichment demonstrates the top STRING interaction network for the gene (accessed on 25 July 2024) (Figure 11G–I).

## 3. Discussion

Despite breakthroughs in therapeutic modalities, GBM patients have a poor prognosis, with a median OS of fewer than 15 months [36]. The molecular and epigenetic heterogeneity of GBM poses a major challenge for diagnosis and therapy [39]. New diagnostic and prognostic indicators, as well as treatment targets, are desperately needed for GBM [40].

DNA methylation is one of the most important epigenetic alterations, regulating gene expression and influencing a variety of cellular processes such as DNA repair, cell cycle, apoptosis, and invasion [17]. Aberrant promoter methylation patterns of tumor suppressor genes and oncogenes have been implicated in cancer pathogenesis and prognosis [18]. The current study sought to investigate the promoter methylation status of four genes (MGMT, NUPR1, NDRG2, and GLI1) that are usually involved in diverse aspects of GBM biology, such as DNA repair, stress response, and signaling [41,42]. The correlation of these genes with each other and with clinical outcomes in GBM patients was explored, with the hypothesis that these genes may serve as potential epigenetic markers for GBM.

To the best of our knowledge, this is the first study to investigate the combined effect of MGMT, NUPR1, NDRG2, and GLI1 promoter methylation on GBM patients’ outcome. While promoter methylation levels of NUPR1 and GLI1 genes had not been previously investigated in this context, our findings revealed that promoter hypermethylation of NUPR1 and MGMT genes may confer a better prognosis to GBM patients, while promoter hypermethylation of GLI1 and NDRG2 genes may confer a worse prognosis to GBM patients.

The promoter methylation status of the MGMT gene was significantly higher in NND samples than in GBM samples. A previous study demonstrated that promoter hypermethylation of the MGMT gene has been connected to a number of non-neoplastic CNS illnesses [43]. This is in line with the results of the present study, which showed that promoter hypermethylation of the MGMT gene was found in all NND samples and in approximately 50% of the GBM samples. Consistent with the results of the current study, a prior study revealed that hypermethylation-induced MGMT epigenetic silencing is present in about 40% of primary glioblastomas [44]. This differential methylation pattern suggests that while dysregulation of MGMT methylation is a hallmark of certain neoplastic processes [45], it may also serve as a biomarker for various non-neoplastic CNS disorders [43]. The prevalence of MGMT promoter hypermethylation in non-neoplastic diseases could reflect a broader epigenetic landscape influencing cellular responses to stress or injury, as supported by findings that suggest altered methylation patterns can impact gene expression and cellular functions in a variety of neurological conditions [46]. Elucidating the disease-specific differential epigenetic patterns is crucial for enhancing the early diagnosis of various types of neoplastic lesions [47]. As a consequence, this approach may be valuable for distinguishing between neoplastic and non-neoplastic lesions through their distinct methylation profiles.

The current study revealed that the promoter methylation status of the NUPR1 gene was significantly reduced in GBM samples compared to NND samples. The NUPR1 gene plays a pivotal role in the development and progression of various types of malignancies, including glioblastomas [48]. In glioblastomas, NUPR1 contributes to tumor growth and resistance to conventional therapies by upregulating genes associated with mitochondrial function and antioxidant responses [49]. This makes NUPR1 a potential key regulator gene in GBM, as its function could induce the metabolic adaptations that allow glioblastoma cells to thrive [50]. Previous studies revealed that GBM tissues showed increased expression of NUPR1 mRNA compared to normal tissues [50]. Empirical evidences indicate that a decrease in methylation levels at the promoter region of a gene leads to an upregulation of the gene’s mRNA and protein expression, resulting in the gene’s enhanced functional activity [51]. As a consequence, the promoter hypomethylation of the NUPR1 gene might increase NUPR1 mRNA and protein levels, enhancing gene expression. The current study revealed that the promoter region of the NUPR1 gene was hypomethylated in 82.2% of GBM samples. These results suggest that promoter hypomethylation-induced NUPR1 gene overexpression may play a role in gliomagenesis and GBM development. Moreover, the oncogenic properties of NUPR1, as evidenced by its involvement in promoting cell growth and metastasis in various cancers [52], indicate that it may serve as a viable biomarker for aggressive tumor behavior and a potential therapeutic target. A recent study has suggested that inhibiting NUPR1 activity could sensitize tumor cells to chemotherapeutic agents, thereby improving treatment efficacy [53].

On the other hand, the promoter methylation status of the NDRG2 gene was significantly higher in GBM samples than in non-neoplastic samples. A previous study revealed that promoter hypermethylation of the NDRG2 gene is the primary mechanism for downregulating NDRG2 expression in GBM [25]. This finding is consistent with the function of NDRG2 as a tumor suppressor, and its downregulation may contribute to the aggressive nature of glioblastomas [54]. The increased promoter methylation of the NDRG2 gene in GBM may disrupt its protective functions, allowing for unchecked cellular proliferation and survival [55]. Additionally, the promoter methylation status of the NDRG2 gene could serve as a potential biomarker for GBM prognosis, as higher levels of methylation may correlate with poorer outcomes [56]. The study also demonstrated that the promoter methylation status of the GLI1 gene was significantly higher in GBM samples than in non-neoplastic samples. The elevated promoter methylation status of the GLI1 gene in GBM samples suggests a crucial role for epigenetic regulation in the pathogenesis of glioblastoma. GLI1 is a key transcription factor in the HH signaling pathway, which is known to be aberrantly activated in various cancers, including GBM [57]. This hypermethylation may lead to altered expressions of the GLI1 gene, thereby disrupting normal HH signaling and contributing to tumor development and progression. Furthermore, GLI1 facilitates tumor aggressiveness by orchestrating the tumor microenvironment (TME); it drives angiogenesis through the direct transcriptional upregulation of vascular endothelial growth factor (VEGFA) and Angiopoietin-2 (ANGPT2), essential for the hyper-vascularized nature of GBM [58]. Simultaneously, GLI1 promotes immune evasion by inducing the secretion of immunosuppressive mediators, such as TGF-β and IL-10, as well as the chemokine CCL2 [59]. These factors collectively facilitate the recruitment of Myeloid-Derived Suppressor Cells (MDSCs) and the pro-tumorigenic M2 polarization of macrophages, effectively shielding the tumor from cytotoxic immune responses, which are critical for GBM aggressiveness [60,61]. Understanding the implications of GLI1 promoter methylation not only sheds light on its role in GBM biology but also presents potential therapeutic avenues. Therefore, targeting the HH pathway or reversing GLI1 hypermethylation could enhance treatment responses.

The present study also demonstrated a significant age disparity between GBM and NND patients (*p* < 0.001). GBM patients were notably older, with nearly one-third (27.6%) being aged 60 or over, while none of the NND participants fell into this age category. This age-related pattern is consistent with a prior study [62], highlighting the critical role of age-related genomic alterations as a critical drive of GBM development. Declining DNA repair mechanisms and impaired cellular senescence pathways likely contribute to this age-associated vulnerability [63].

The current study revealed that MGMT/NUPR1 promoter hypomethylation and NDRG2/GL1 promoter hypermethylation correlate with GBM evolution, suggesting that they may play contrasting roles in GBM pathogenesis. MGMT/NUPR1 methylation loss could enable tumorigenesis, whereas NDRG2/GL1 methylation activation may promote GBM progression. The contrasting methylation patterns of MGMT/NUPR1 and NDRG2/GLI1 in GBM may shed light on their opposing roles in tumor development and progression. The study also revealed a strong positive correlation in the promoter methylation status of the MGMT and NUPR1 genes, suggesting that they may be epigenetically co-regulated. This co-regulation could potentially occur through similar transcription factor binding sites in their promoter regions. Understanding these shared regulatory elements could provide insight into coordinated gene expression. According to an earlier study, the NUPR1 gene modulates the effect of Kirsten rat sarcoma viral oncogene homolog (KRAS G12D)-induced senescence via controlling DNMT1 expression and, as a result, genome-wide levels of DNA methylation [64]. Additionally, KRAS mutations have been linked to MGMT methylation level [65]. As a consequence, the epigenetic aberration rather than a causal relationship could underlie the association between MGMT methylation, KRAS mutation, and the NUPR1 methylation network. This association is likely mediated by mutant KRAS-induced activation of the MAPK/ERK pathway, which modulates the activity of downstream effectors, such as activator protein 1 (AP-1) and activating transcription factor 6 (ATF6) [66]. These transcription factors are known to recruit the epigenetic machinery, including DNA methyltransferases (DNMTs), to shared regulatory elements within the promoter regions of the NUPR1 and MGMT genes [67,68] Consequently, the hypermethylation of MGMT represents a downstream epigenetic consequence of KRAS-mediated signaling aberrations, which disrupts chromatin accessibility and transcriptional activity, rather than MGMT exerting feedback control over KRAS expression [69,70]. These findings suggest a potential mechanism for the coordinated regulation of NUPR1 and MGMT expression, with implications for cellular processes and disease states.

The current study also revealed positive correlations between the promoter methylation status of the NDRG2 gene and the GLI1 gene. Previous studies have linked the suppression of NDRG2 expression in tumor cells to hypermethylation, which is primarily controlled by DNMTs [71,72]. A key risk factor for enhanced DNMT activity was high ROS [73]. The formation of ROS triggers the activation of GLI1 signaling and regulates the proliferation and apoptosis of tumor cells [74]. Furthermore, the hypermethylation of the NDRG2 promoter has been shown to be an early event in glioma development, contributing to the aggressive nature of these tumors [75]. The interplay between ROS and GLI1 signaling not only affects tumor cell survival but also influences the TME, promoting angiogenesis and immune evasion [76]. The current study revealed negative correlations between the promoter methylation status of the MGMT/NUPR1 gene and the NDRG2/GLI1 gene, suggesting potential reciprocal regulation between these two gene groups. A prior study demonstrated that GLI1 binding to the MGMT promoter region affects its expression, indicating that MGMT is a downstream target gene of the HH/GLI1 signaling pathway [77]. Hypermethylation and silencing of MGMT/NUPR1 may allow for increased expression and decreased methylation levels of the latter genes and vice versa. The correlation patterns among these tumor suppressors and oncogenes may hint at an epigenetic reprogramming event in GBM tumorigenesis that co-modifies critical gene regulatory networks.

The present study revealed that the promoter regions of the MGMT and NUPR1 genes were more frequently methylated in GBM patients who achieved CR or PR compared to those with SD or PD. This suggests that the methylation status of these genes may serve as predictive markers for a favorable therapeutic response in GBM patients. Previous studies have linked MGMT promoter methylation to decreased MGMT expression and enhanced susceptibility to TMZ [78,79]. NUPR1 expression was lowered as a result of hypermethylation [80], and treatment responsiveness improved. This is due to the evidence that NUPR1 promotes glioma cell autophagy and TMZ resistance via the KDM3A/TFEB axis [50]. As a result, the promoter methylation status of MGMT and NUPR1 could be employed as methylation biomarkers to predict the response and fate of TMZ-treated GBM patients. The integration of multiple methylation markers not only enhances the predictive accuracy of prognostic models but also underscores the complex interplay between genetic and epigenetic factors in GBM. The differential methylation status of MGMT and NUPR1 genes in patients with varying therapeutic responses highlights the potential of these markers in stratifying patients for personalized treatment regimens. The study also revealed that the promoter status of NDRG2 and GLI1 genes was more frequently hypermethylated in patients who had PD compared to patients who achieved CR or PR. This suggests that promoter hypermethylation of these genes may be predictive of poor response to therapy in GBM patients. A prior study demonstrated that hypermethylation of the NDRG2 promoter region is significantly associated with increased tumor aggressiveness and resistance to standard therapies [24]. As the grade of the glioma increases, NDRG2 expression decreases, which is associated with a worse overall prognosis [54]. A prior investigation found a correlation between high GLI1 mRNA levels and a poor prognosis for both OS and PFS [77,81]. Additionally, GLI1, a key transcription factor in the HH pathway, has been implicated in the maintenance of glioma stem cells, which are known to contribute to tumor recurrence and therapeutic resistance [42].

Cox regression analysis identified NUPR1 promoter methylation status as an independent prognostic biomarker for OS in GBM patients. Hypermethylation of the NUPR1 promoter, which may downregulate gene expression, was associated with improved OS. This positive correlation suggests that promoter hypermethylation may suppress NUPR1 expression, leading to better survival outcomes. This finding aligns with a previous study by Murphy and Costa [28], which reported that increased NUPR1 expression is associated with poor survival outcomes. Kaplan–Meier survival analysis provided additional insights, illustrating that patients with elevated promoter methylation levels of MGMT and NUPR1 genes experienced significantly longer PFS compared to those with low promoter methylation levels. In contrast, patients exhibiting high levels of promoter methylation in NDRG2 and GLI1 genes showed reduced PFS, reinforcing the idea that these methylation patterns are intricately linked to disease progression and patient outcomes.

The study also looked into the potential use of DNA methylation markers in GBM patients. ROC analysis was utilized to investigate NUPR1, MGMT, NDRG2, and GLI1 methylation’s discriminative, prognostic, and predictive abilities. It is accepted, per our group previous research work, that the optimal level threshold for genes to be identified to achieve the best balance between sensitivity and specificity for predicting disease/cancer and outcomes is either survival or therapeutic response [82,83,84,85,86]. Therefore, in the current study, these thresholds can be used to develop “methylation-based prognostic tool(s)”. In terms of discriminatory ability, all four genes had excellent discriminative capacity between GBM and NND groups, with AUC values of more than 0.75. NUPR1 had the highest AUC (0.858), followed by NDRG2 (0.973), MGMT (0.793), and GLI1. These findings suggest that promoter methylation levels of these genes can efficiently discriminate between GBM and non-neoplastic conditions. The study also found that all four genes have considerable prognostic value. Promoter hypermethylation levels of NUPR1 and MGMT genes demonstrated the highest AUC values, approaching 0.80, which indicates their robust capacity to predict disease progression in GBM patients. While promoter methylation levels of NDRG2 and GLI1 genes exhibited acceptable AUC values (ranging from 0.76 to 0.79), they nevertheless provided valuable prognostic information. Furthermore, all four genes demonstrated promising predictive abilities regarding treatment response. Once again, NUPR1 and MGMT stood out, with AUC values between 0.83 and 0.84, suggesting a strong ability to forecast a patient’s response to therapeutic interventions, particularly in relation to alkylating agents, like TMZ. NDRG2 and GLI1 also showed commendable predictive performance, with AUCs exceeding 0.75. This data can inform decisions about the continuation, modification, or escalation of therapeutic regimens based on predicted outcomes.

It is worth mentioning that the objective of this research was to identify an independent epigenetic signature that functions across the clinical spectrum of GBM. Statistically, the methylation status of our four-gene panel demonstrated high significance as a predictor of survival and treatment response, suggesting that these specific epigenetic changes represent a downstream biological behaviour that may be independent of the IDH mutation pathway (the most important marker for diffuse astrocytic tumors). The high statistical significance observed in our cohort, without IDH stratification, indicates that these epigenetic alterations are robust indicators of the “final common pathway” of GBM aggressiveness. Therefore, this panel offers a valuable, simplified molecular tool for predicting clinical outcomes that remains informative across the histological spectrum of GBM.

**Figure 12:**
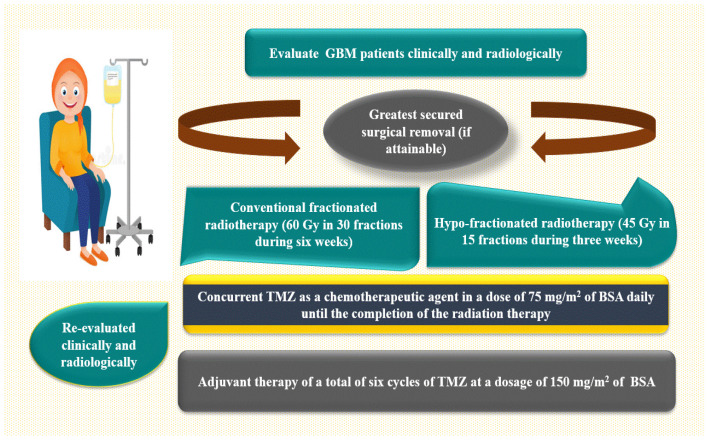
GBM patient therapeutic strategy infographic. [GBM: glioblastoma, Gy: gray, TMZ: temozolomide, BSA: body surface area].

In summary, the findings of this study underscore the promising discriminative, prognostic, and predictive capabilities of the promoter methylation levels of NUPR1, MGMT, NDRG2, and GLI1 genes in GBM. These methylation markers may serve as potential biomarkers for risk stratification, prognosis assessment, and treatment prediction within the studied population. The integration of these epigenetic markers, being part of our research group’s search into the genetic/epigenetic of various diseases [87,88,89,90,91,92,93], into clinical settings could lead to improved patient outcomes through more tailored therapeutic strategies, ensuring that patients receive the most effective therapies based on their unique genetic/epigenetic profiles. All these findings were confirmed by functional enrichment analysis, where MGMT is related to NUPR1 or GLI1 as well as NDRG2 indirectly via CDK2, TP53, or Myc. Moreover, each could be a therapeutic target treatment, as predicted and suggested by in silico bioinformatics UCSC analysis treatment to the latter genes/proteins (Figure 12) targeting CDK2 by bosutinib and TP53 by acetyl salicylic acid. Limitation(s). The study acknowledges the limitations of the sample size with limited demographic diversity. Future research with more diverse cohorts and multi-center collaboration is recommended to validate and extend these findings to be integrated in the prognostic, predictive, and personalized (3P) [94] “Genome Project” after integrating next-generation sequencing and/or CRISPR-based gene editing. Moreover, testing the potential synergy between the current panel focusing on a specific epigenetic signature related to stress response and DNA repair pathways and CDKN2A status, a known marker of aggressiveness, remains a subject for future investigation.

## 4. Materials and Methods

### 4.1. Sample Size and Study Power

Based on the previous study by Kolodziej et al. in 2016 [25], the number of cases required to attain a type one error alpha less than 0.05 and a power of 95% by the Mann–Whitney U test is 58 samples using G*power output version 3.1.9.4 for Windows 10.

### 4.2. Study Design

Case–Controlled Prospective Mono-Center Study.

### 4.3. Study Clinical Trial Registration

ClinicalTrials.Gov Identifier: NCT06538428.

### 4.4. Institutional Review Board (IRB) Statements

The study protocol was approved by the Research Ethics Committee of the Faculty of Pharmacy, Ain Shams University, Cairo, Egypt, serial No. ENREC-ASU 2020-8, and the Medical Ethical Committees at the National Research Center ID#20110. Study participants were recruited during the period from September 2020 to October 2022, after being informed of the study’s problem, aim, and objectives. A written REC-approved informed consent (I.C) form was signed by study participants or their first-degree relatives. The study adhered to the ethical principles outlined in the Declaration of Helsinki 2013 [95].

### 4.5. Study Participants

#### 4.5.1. Patients Group

A cohort of fifty-eight treatment-naïve patients with a confirmed diagnosis of de novo grade 4 glioma was enrolled from the Clinical Oncology Unit, Faculty of Medicine, Ain Shams University Hospital, Cairo, Egypt.

##### Patients’ Inclusion Criteria

Inclusion criteria were adult patients (over the age of eighteen) with a recent diagnosis of grade 4 glioma and an overall performance score of below or equal to 2 on the Ester Clinical Oncology Group (ECOG) scale: https://ecog-acrin.org/resources/ecog-performance-status/. The ECOG scale measures how the disease is progressing and how it affects patients’ ability to function on a day-to-day basis [96]. A score of 1 means that patients are limited in physically demanding activities yet mobile and capable of performing light or sedentary tasks, while a score of 2 means that patients are ambulatory, awake for more than half of their waking hours, and capable of all self-care, but they are unable to perform any work-related tasks.

##### Therapeutic Approach

Clinical evaluations have been performed on all GBM patients, involving a comprehensive medical history, physical examinations, and neurological assessments. The patient underwent brain imaging to facilitate the administration of a standard therapeutic protocol, as depicted in Figure 12. This protocol includes the most secure surgical excision (if possible), followed by standard fractionated radiotherapy (FRT) [aggregate dose of 60 gray (Gy), provided 2 Gy per fraction for 30 fractions during six weeks] or hypo-fractionated radiotherapy [45 Gy in 15 fractions during three weeks] in conjunction with concurrent TMZ as a chemotherapeutic drug at a dose of 75 mg/m^2^ of body surface area daily (BSA). The patient was then re-evaluated clinically and radiologically, and an adjuvant therapy was administered at a dose of 150 mg/m^2^ of BSA from day one to day five, for a total of 28 days, under close medical surveillance [97,98,99]. During the context of conventional clinical surveillance, patients were evaluated using gadolinium-enhanced magnetic resonance imaging (Gd-MRI) after 45 days following radiotherapy and subsequently every three months or whenever clinical evidence of neurological deterioration was noticeable.

Tumor response was assessed using radiological Response Assessment in Neuro-Oncology (RANO) response criteria, as shown in Table 8 [100]. The absence of all documented tumor lesions is referred to as a complete response (CR). A partial response (PR) is defined as a 50% or higher reduction in precalculated tumor lesions or a quantifiable enhancement of pre-assessed tumor lesions. Stable disease (SD) is defined as no changes in the size of tumor lesions (either <50% decline or <25% increase in the size of premeasured tumor lesions). Progressive disease (PD) is defined as a ≥25% rise in the dimensions of some or all brain lesions, as well as the emergence of any additional brain lesions.

##### Patients’ Demographic, Clinical, and Pathological Data

The demographics of fifty-eight GBM patients were retrieved from the hospital’s medical records, including age, gender (35 male and 23 female), clinicopathological features, such as ECOG score, time of initial GBM surgery, the degree of the tumor dissection, previously identified therapeutic strategy, and survival data [progression-free survival (PFS) and overall survival (OS)]. PFS denotes the duration between patient enrolment in the study and the onset of disease progression. In addition to the comprehensive medical or surgical history of the patient regarding GBM, the entire familial history of GBM and any prior records of other malignancies were documented. The size (in centimeters) and location (in multiple cerebral lobes, the right or left) of the tumor were also determined.

#### 4.5.2. The Non-Neurooncological Diseases (NND) Group

Twenty sex-matched participants with focal cortical dysplasia (FCD) and a left parieto-occipital space-occupying lesion constitute the NND group in a comparable male-to-female ratio of 1:1 (to minimize potential sex-related epigenetic variability within the NND cohort) and an age range of 19 to 54 years. Individuals had been selected at random and had no previous history of mailgnancy.

### 4.6. Methods

#### 4.6.1. In Silico Database(s) Search and Bioinformatics Analysis (Accessed 17 October 2023 and Revised July 2024)

##### Differential Gene Expression (DGE) of Different Genes from Online Datasets in GBM

To retrieve relevant gene expression data, we accessed UCSC Xena Browser [101], https://xenabrowser.net (accessed on 25 July 2024), and selected the dataset (TCGA Glioblastoma (GBM)) from the active data hub, which consists of 25 datasets, followed by visualization. The first variable is the phenotypic data type. The basic phenotype is a sample type that is the primary tissue vs. the normal solid tumor, which is used to compare the expression of different genes in available online datasets via the Xena DGE Analysis Pipeline, adapted from bulk RNA-seq analysis and further downstream analyses. The second variable is the four genes of interest. This is followed by principal component analysis (PCA) as a form of quality control (QC).

##### Pathway Enrichment Analysis (PEA)

To determine whether a set of genes shows statistically significant, non-random association using the Kyoto Encyclopedia of Genes and Genomes (KEGG) database in GBM was used.

##### Identification of the Investigated Genes by Bioinformatics (Accessed 17 October 2023 and Revised July 2024)

The ENCODE portal [102], https://www.encodeproject.org/, EMBL’s European Bioinformatics Institute EMBL-EBI of big data in biology, https://www.ebi.ac.uk/ the Human Genome Research Institute grant supporting the HUGO Gene Nomenclature Committee (HGNC), https://www.genenames.org/, the National Center for Biotechnology Information (NCBI), https://www.ncbi.nlm.nih.gov/ (USA.gov), and Ensembl Databases, https://www.ensembl.org/index.html (accessed on 17 October 2023), were used to examine gene characterization, as shown in Table 11.

Human MicroRNA Disease Database (HMDD) v4.0 June 2023 for retrieving diseases similar to GBM from the disease network database, http://www.cuilab.cn/hmdd (Accessed Revised 23 July 2024) [103].

##### Functional Enrichment Analysis, Targeted Pathways, and Heatmaps

KEGG targeted pathways, https://www.genome.jp/kegg/pathway.html, for glioma/GBM genetic alteration pathways (accessed on 17 October 2023) were used.

Functional enrichment analysis used STRING version 11.5, https://string-db.org/ (Accessed Revised 27 July 2024) [104]. Finally, we used the Genome Browser (UCSC) [105] (December 2013, initial release; June 2022, patch release 14; selected top genes). Targets of the examined gene interactions (protein–protein interaction (PPI) or gene–gene interaction) and pathways from the curated databases and text mining, https://genome.ucsc.edu/cgi-bin/hgGateway (accessed on 25 July 2024), were used.

#### 4.6.2. Sample Processing

Brain tissue samples were surgically obtained via an open stereotactic biopsy approach, kept in neutral buffered formalin, and embedded in paraffin labeled with hematoxylin–eosin (H&E) prior to the administration of any treatments. The diagnosis was subsequently confirmed by a panel of neuropathologists evaluating all enrolled samples in compliance with the 2016 WHO classification of CNS tumors [106,107].

##### Optimizing GBM Tissue Quality for Accurate DNA Methylation Profiling

The GBM tissue chunks were selected based on the eligibility requirements as follows: the histopathological verification of GBM tissue with at least 80% viable malignant cells, along with the availability of archival paraffin-embedded tissue sections. The following techniques were performed on all GBM tissues. A full slice of the formalin-fixed, paraffin-embedded (FFPE) chunk of GBM tissues was cut at a depth of 4 μm and then dyed with standard HE stain to assess the viability of the provided neoplastic tissue. Samples for the PCR procedure were prepared using freshly cut FFPE slices up to 10 μm in thickness. Each preparation can contain up to 8 slices, all of which have a maximum thickness of 10 μm and a maximum surface area (SA) of 250 mm^2^. Then, 5–10 FFPE sections were transferred to Eppendorf tubes for further DNA extraction processing.

#### 4.6.3. DNA Extraction

The DNA was retrieved from FFPE specimens using a QIAamp FFPE kit (Cat. No. 56404, Valencia, CA, USA), as indicated by the supplier. The concentration and purity were assessed using a nono-drop spectrophotometer (Quawell, Q-500, Scribner, MI, USA) by measuring the magnitude of absorbance at 260 and 280 nm and validated on a 1% agarose gel. The extracted DNA specimens were stored at −20 °C for further investigation to determine promoter methylation levels of the MGMT, NUPR1, NDRG2, and GLI1 genes.

##### Detection of Promoter Methylation Patterns of MGMT, NUPR1, NDRG2, and GLI1 Genes Using Methyl II Quantitative PCR

The EpiTect Methyl II quantitative polymerase chain reaction (qPCR) technology (Qiagen, Germany) has been used to identify promoter methylation profiles of the MGMT, NUPR1, NDRG2, and GLI1 genes in DNA-retrieved samples. This was verified by employing restriction enzymes specific to methylation to measure the quantity of extracted DNA that is left behind after fragmentation. The remaining DNA was quantified by real-time PCR using specific methylation primers for the targeted genes that contain promoter regions of interest.

##### Methylation-Specific Restriction Enzyme Digestion Technique

The procedure was conducted in two phases. In Phase I, the EpiTect Methyl II DNA Restriction Kit (cat. no. 335452, Foster City, CA 94404, USA) was utilized for the reactions. Genomic DNA, which had been previously extracted, was aliquoted into four equivalent portions in four separate PCR reaction tubes. One tube (MO) had no added restriction enzyme, the second tube (Ms) had a methylation-sensitive restriction enzyme (MSRE) that selectively digests unmethylated DNA, the third tube (Md) had a methylation-dependent restriction enzyme (MDRE) that digests methylated DNA, and the fourth tube (Msd) had both MSRE and MDRE, which were utilized to assess the background and proportion of input DNA that was resistant to digestion by enzymes. The tubes were then subjected to incubation in a thermal cycler (SureCycler 8800, Agilent, Santa Clara, CA, USA) at 37 °C for 6 h, followed by incubation at 65 °C for 20 min.

##### Quantitative PCR (qPCR) Analysis

The quantification of the remaining genomic DNA samples in each tube was performed in Phase II using a Max3005P qPCR system (Stratagene, Agilent Technologies, La Jolla, CA, USA). To initiate this phase, a 5 μL aliquot of the remaining DNA from each tube was mixed with the qPCR master mix (RT2 qPCR SYBR Green/ROX Master Mix, Cat. no. 330520, Santa Clarita, CA, USA). The resulting mixture was then dispensed into a PCR plate containing pre-aliquoted MGMT, NUPR1, NDRG2, and GLI1 methylated and unmethylated primers. The primer sequences used are listed in Table 12 and were retrieved from https://www.primer3plus.com/. The relative portions of methylation and unmethylated DNA were calculated by comparing the levels in each digest to those in a mock digest (without enzymes) using the ΔCT method. Calculations were performed by collecting the raw ΔCT values for each PCR reaction tube (UM and M) for every sample. The methylation percentage was calculated using the following equation: methylation % = 2 − ΔCT fold change  × 100.

### 4.7. Statistical Analysis

The Shapiro–Wilk and Kolmogorov–Smirnov tests were used to test the normality of the data. Regarding descriptive statistics, frequencies (*n*) and percentages (%) were used to represent categorical variables. The mean (M) ± standard deviation (S.D) was used to describe normally distributed continuous variables, whereas the median and interquartile range [25th–75th percentile] [IQR] were used to describe not normally distributed continuous variables. The Mann–Whitney U test was utilized to compare the methylation levels of the MGMT, NUPR1, NDRG2, and GLI1 genes between the NND and GBM groups and the methylation status of the four genes in each patient of the two groups. The optimal cut-off points for promoter methylation of the four genes were determined after being plotted on receiver operating characteristic (ROC) curves using the mean percentage of methylation in each patient. The area under the curve (AUC) and test accuracy were calculated. The likelihood ratio (LR) as a measure of diagnostic accuracy, sensitivity (SN), specificity (SP), and accuracy was calculated for several cut-off points. The probability of a person having a positive disease test divided by the probability of the same person not having the disease is expressed as LR+. The higher the LR+ value, the more informative the diagnostic test is. Survival data (in months) were calculated from the start of each chemotherapy line to the date of death or last follow-up. Progression-free survival (PFS) is the period that elapsed between the beginning of patient recruitment and the onset of disease progression, as determined by MRI scans or clinical decline. Overall survival (OS) was estimated between the time of random allocation and the time of death or censored at the time of the last follow-up. Differences in PFS and OS were compared using Kaplan–Meier survival curves, and the log-rank test was used to determine statistical significance. The Cox proportional hazards model was used for multivariate survival analysis. It identifies factors that are likely to have a significant impact on OS. Cox regression multivariate analysis was performed using a forward stepwise parameter with a significance of 0.05 for entry and 0.1 for removal of factors significantly associated with OS. The binary logistic regression model was used to analyze the prognostic significance of the methylation of the four genes, MGMT, NUPR1, GLI1, and NUPR1, as well as sex, age, tumor size, and ECOG with respect to clinical outcomes. The goodness of fit of the logistic regression models was evaluated using the Hosmer–Lemeshow test. A significance level of a *p* value of less than 0.05 was chosen. Statistical analysis was performed using the statistical package for social studies software (SPSS) version 27.0 (IBM, Armonk, NY, USA), MedCalc^®^ Statistical Software 2023 version 22.009 (MedCalc Software Ltd., Ostend, Belgium), https://www.medcalc.org/en/, R Core Team (2023) version 4.3.2, R: A Language and Environment for Statistical Computing R Foundation for Statistical Computing, Vienna, Austria, https://www.R-project.org/, and GraphPad Prism version 9.5.1 for Windows, GraphPad Software, San Diego, CA, USA, www.graphpad.com.

## 5. Conclusions

Integrated analysis of multiple methylation molecular markers enabled the construction of prognostic models with improved predictive accuracy over the individual biomarkers for GBM. Our current study identified the clinical usefulness of MGMT, NUPR1, NDRG2, and GLI1 promoter methylation as promising, predictive, and/or prognostic molecular biomarkers as well as potential epigenetic therapeutic targets in GBM patients. This approach moves beyond a single-gene biomarker (MGMT) towards a more comprehensive model that reflects the molecular heterogeneity of GBM. The strong correlations and contrasting prognostic values that we found between these genes (e.g., MGMT/NUPR1 vs. NDRG2/GLI1) validate this integrative strategy and suggest an interconnected epigenetic landscape in GBM. The current study is the first to evaluate these four genes together as an epigenetic signature panel for diagnosis, prognosis, and therapy response prediction in GBM. This holistic view allows for a more robust and nuanced understanding of GBM epigenetics. NUPR1 could serve as an independent prognostic factor, which represents a novel contribution to the field, especially given its strong correlation with MGMT and its potential role in TMZ response. In addition, multivariate models were constructed, incorporating methylation status, clinical variables, and treatment outcomes, which showed strong discriminatory ability and identified NDRG2 hypermethylation as an independent predictor of GBM. These models, together with defining optimal methylation cut-offs for each gene to stratify patients into risk groups, hold promise for clinical translation toward personalized therapy.

## Figures and Tables

**Table 1:** Demographic characteristics of the NND (*n* = 20) and GBM (*n* = 58) groups.

Patient Characteristics (Unit)		Group (*n*)		
		NND (20)	GBM (58)	*p* Value
Age (median [IQR])		28.50 [24.50, 36.00]	51.00 [39.00, 58.00]	<0.001 *
Age group	<60 (*n*, %)	(20, 100%)	(42, 72.4%)	=0.008 ^
	≥60 (*n*, %)	(0, 0%)	(16, 27.6%)	
Gender (*n*) (%)	Female	10 (50%)	23 (39.7%)	NS ^
	Male	10 (50%)	35 (60.3%)	
MGMT (median [IQR])		98.00 [97.00, 100.00]	66.00 [13.00, 97.00]	<0.001 *
NUPR1 (median [IQR])		98.00 [95.00, 100.00]	65.00 [23.00, 87.00]	<0.001 *
NDRG2 (median [IQR])		19.00 [12.50, 21.50]	67.00 [54.25, 75.00]	<0.001 *
GLI1 (median [IQR])		20.00 [12.75, 22.00]	40.50 [26.25, 55.50]	<0.001 *
ECOG status (*n*) (%)	1	-	19 (32.8%)	
	2	-	39 (67.2%)	
Tumor site (*n*) (%)	Left	-	29 (50%)	
	Right	-	23 (39.7%)	
	Multiple	-	6 (10.3%)	
Tumor size (%)	<5 cm	-	22 (37.9%)	
	≥5 cm	-	36 (62.1%)	
GBM family history (%)	No	-	45 (77.6%)	
	Yes	-	13 (22.4%)	
Surgical intervention	Biopsy	-	36 (62.1%)	
	GTR	-	19 (32.7%)	
	STR	-	3 (5.2%)	
Therapeutic response	CR	-	15 (25.9%)	
	PR	-	8 (13.8%)	
	SD	-	7 (12.1%)	
	NR	-	28 (48.3%)	
PFS (months) (median [IQR])		-	6.00 [4.00, 19.00]	
GBM progression	yes	-	31 (53.4%)	
	No	-	27 (46.6%)	
OS (months) (median [IQR])		-	10.00 [6.00, 23.00]	
Status	Alive	-	35 (60.3%)	
	Died	-	21 (36.2%)	
	Lost to follow-up	-	2 (3.4%)	

Data are presented as median [interquartile range] for continuous variables and number (percentage) for categorical variables. Data generated using R software (version 4.3.2) and the tableone package (version 0.13.2). Statistical analyses were performed using the ^ Chi-square test (χ^2^) for categorical variables and the * Mann–Whitney U test (U) for continuous variables. The level of significance *p* value was set at 0.05. [MGMT: O6-methylguanine-DNA methyltransferase, NUPR1: Nuclear Protein 1, NDRG2: N-Myc Downstream-Regulated Gene 2, GLI1: glioma-associated oncogene homolog 1, ECOG: Eastern Cooperative Oncology Group, GTR: gross total resection, STR: subtotal resection, CR: complete response, PR: partial response, SD: stable disease, PFS: progression-free survival, OS: overall survival, NS: non-significant].

**Table 2:** Promoter methylation patterns of MGMT, NUPR1, NDRG2, and GLI1 genes among the NND (*n* = 20) and GBM (*n =* 58) groups and within each group.

Genes Methylation Status	Groups (*n*)		Statistics
Unit *n* (%)	NND (*n* = 20)	GBM (*n* = 58)	*p* Value
MGMT			
<66% (low methylation)	0 (0%)	28 (48.3%)	χ^2^ = 15, *p* < 0.0001
≥66% (high methylation)	20 (100%)	30 (51.7%)	
NUPR1			
<96.5% (low methylation)	5 (25%)	50 (86.2%)	χ^2^ = 26.7, *p* < 0.001
≥96% (high methylation)	15 (75%)	8 (13.8%)	
NDRG2			
<32.5% (low methylation)	19 (95%)	2 (3.5%)	χ^2^ = 63.3, *p* < 0.001
≥32.5% (high methylation)	1 (5%)	56 (96.5%)	
GLI1			
<24% (low methylation)	20 (100%)	13 (22.4%)	χ^2^ = 36.6, *p* < 0.001
≥24% (high methylation)	0 (0%)	45 (77.6%)	

Data generated using SPSS software (version 27.0). Methylation status of MGMT, NUPR1, NDRG2, and GLI1 genes was categorized as low or high based on a predetermined threshold for each gene using ROC analysis. Statistical analyses were performed using the chi-square test (χ^2^) for categorical variables. The level of significance *p* value was set at 0.05. [NND: non-neurooncological disease, GBM: glioblastoma, MGMT: O6-methylguanine-DNA methyltransferase, NUPR1: Nuclear Protein 1, NDRG2: N-Myc Downstream-Regulated Gene 2, GLI1: glioma-associated oncogene homolog 1].

**Table 3:** Associations between GBM patients’ (*n* = 58) clinical characteristics and the MGMT, NUPR1, NDRG2, and GLI1 gene methylated patterns.

Clinical	Gene Methylated Patterns (Median [IQR])			
Characteristics (Unit)	MGMT	NUPR1	NDRG2	GLI1
Age (years)				
<60	66 [13, 97]	76 [27, 92]	65 [54, 75]	40.5 [25, 56]
≥60	19 [13, 81]	29 [23, 81.5]	73 [65, 76]	42 [27, 55]
Statistics *	NS	NS	NS	NS
Gender: M, F				
Male	34 [14, 66]	44 [24, 84]	57 [52, 75]	44 [23.5, 56]
Female	97 [13, 97]	77 [23, 95.7]	69 [65.2, 76.7]	40 [30.7, 48]
Statistics *	NS	NS	NS	NS
Pathology				
ECOG = 1	97 [66, 100]	77 [76, 97]	55 [52.8, 58.7]	21 [16, 34.2]
ECOG = 2	19 [13, 97]	29 [23, 87]	73 [65, 77]	45 [38.5, 65.5]
Statistics *	*p* < 0.001 *	*p* < 0.001 *	*p* < 0.001 *	*p* < 0.001 *
Tumor site				
Left	66 [13, 97]	76 [22.7, 92.2]	65 [52, 77]	38 [22.5, 45.7]
Right	66 [14.5, 97]	67 [29, 91.5]	67 [55.5, 73.7]	40 [26.2, 59.5]
Multiple	16 [13, 19]	25 [23, 29]	73 [72, 79]	65.5 [45, 73]
Statistics ^	NS	NS	NS	*p* = 0.045 ^
Tumor size (cm)				
<5	97 [66, 97]	87 [63, 95]	67.5 [56, 77]	34.5 [25, 40]
≥5	19 [13, 66]	29 [22.5, 76]	65 [53.5, 75]	46.5 [33.5, 66]
Statistics *	*p* = 0.002	*p* = 0.006	NS	*p* = 0.005

Statistics were performed using MedCalc^®^ version 22.009 software utilizing the * Mann–Whitney U test and the ^ Kruskal–Wallis test. The level of significance *p* value was set at 0.05. [MGMT: O6-methylguanine-DNA methyltransferase, NUPR1: Nuclear Protein 1, NDRG2: N-Myc Downstream-Regulated Gene 2, GLI1: glioma-associated oncogene homolog 1, NS: non-significant].

**Table 4:** Relation between therapeutic response and investigated DNA methylation markers.

			Therapeutic Response (*n* = 58)						Test
Methylated	CR (*n* = 15)		PR (*n* = 8)		SD (*n* = 7)		PD (*n* = 28)		Statistics
gene	Mdn	95% CI, 25–75 P	Mdn	95% CI, 25–75 P	Mdn	95% CI, 25–75 P	Mdn	95% CI, 25–75 P	*p*
MGMT	97	97–99.2, 97–99.2	66	66–100, 66–100	66	34–97, 42–89.2	13	13–19, 13 to 19	KWt = 22.2, *p* < 0.001
NUPR1	87	77–97, 77–97	76	66.2–95.4, 71.5–85.5	76	48.9–96.5, 59.5–95	25	23–29, 20–35	KWt = 19.3, *p* = 0.0002
NDRG2	55	53.3–66.5, 53.2–66.5	57	38.8–66.6, 49–61.5	65	42.4–76.5, 52.7–72.5	74	72–77, 67–77	KWt = 12.9, *p* = 0.004
GLI1	36	32.3–44.5, 32.2–44.5	17	12.8–35.1, 13.5–29.5	34	17.5–84, 20–71.7	46.5	44.3–60.5, 41–64.5	KWt = 16.6, *p* = 0.0008

The table was generated using SPSS software (version 27.0). The statistical method used was the Kruskal–Wallis H test (KWt). The level of significance *p* value was set at 0.05. [MGMT: O6-methylguanine-DNA methyltransferase, NUPR1: Nuclear Protein 1, NDRG2: N-Myc Downstream-Regulated Gene 2, GLI1: glioma-associated oncogene homolog 1, CR: complete response, PR: partial response, SD: stable disease, PD: progressed disease].

**Table 5:** Univariate and multivariate Cox regression model for NUPR1, NDRG2, GLI1, age, tumor size, gender, and surgical intervention.

Variables ^#^		HR (Univariable)	HR (Multivariable)
NUPR1		0.97 (0.96–0.99, *p* = 0.002)	0.91 (0.87–0.95, *p* < 0.001)
NDRG2		1.03 (0.99–1.06, NS)	1.00 (0.97–1.04, NS)
GLI1		1.01 (0.99–1.03, NS)	1.01 (0.98–1.05, NS)
Age (years)		0.99 (0.96–1.02, NS)	0.99 (0.94–1.05, NS)
Tumor size (cm)	<5 cm		
	≥5 cm	0.61 (0.25–1.48, NS)	9.81 (2.4 to 39.7, *p* = 0.0014)
Tumor site	Left		
	Right	2.74 (0.97–7.76, NS)	3.20 (0.41–25.11, NS)
	Multiple	4.44 (1.42–13.88, *p* = 0.010)	1.63 (0.25–10.66, NS)
Gender	Male		
	Female	1.02 (0.41–2.55, NS)	0.09 (0.01–0.72, *p* = 0.024)
Surgical intervention	STR	12.12 (2.72–53.93, *p* = 0.001)	223.35 (12.70–3927.93, *p* < 0.001)
	Biopsy		
	GTR	1.07 (0.38–3.04 NS)	3.08 (0.44–21.43, NS)

The table was generated using R software (version 4.3.2), and the packages used were survival, survminer, dplyr, and broom. The statistical methods used were univariate and multivariate Cox proportional hazard ratio (HR). The level of significance *p* value was set at 0.05. ^#^ due to the strong correlation with the NUPR1 gene, MGMT was excluded from the analysis, and a stepwise selection method was employed to enhance the model’s predicted accuracy, resulting in the selection of only three genes. [NUPR1: Nuclear Protein 1, NDRG2: N-Myc Downstream-Regulated Gene 2, GLI1: glioma-associated oncogene homolog 1, STR: subtotal resection, GTR: gross total resection, NS: non-significant].

**Table 6:** Multivariate Cox regression model for NUPR1, NDRG2, GLI1, age, and tumor size.

Covariate ^#^	B	SE	*p* Value	Exp(B)	95% CI of Exp(b)
NUPR1	−0.04457	0.01234	0.0003	0.9564	0.9335 to 0.9798
NDRG2	0.003074	0.01486	NS	1.0031	0.9743 to 1.0327
GLI1	0.0002327	0.01624	NS	1.0002	0.9689 to 1.0326
Age (years)	−0.1620	0.5350	NS	0.8504	0.2980 to 2.4268
Tumor size (cm)	2.2838	0.7131	0.0014	9.8143	2.4258 to 39.7062

The table was generated using SPSS software (version 27.0). The statistical method used was the Cox proportional hazard ratio. The level of significance *p* value was set at 0.05. [NUPR1: Nuclear Protein 1, NDRG2: N-Myc Downstream-Regulated Gene 2, GLI1: glioma-associated oncogene homolog 1, NS: non-significant]. ^#^ due to the strong correlation with the NUPR1 gene, MGMT was excluded from the analysis, and a stepwise selection method was employed to enhance the model’s predicted accuracy, resulting in the selection of only three genes.

**Table 7:** Associations between methylation levels of genes and GBM status: univariate and multivariate analysis.

	OR (95% CI, *p*)	
Variables/Genes	Univariate	Multivariate
NUPR1	0.83 (0.74–0.91, *p* < 0.001)	0.83 (0.58–1.04, NS)
NDRG2	1.45 (1.25–1.85, *p* < 0.001)	1.71 (1.25–3.57, *p* = 0.028)
GLI1	1.26 (1.14–1.45, *p* < 0.001)	1.24 (0.98–1.83, NS)
Age (Years)	1.23 (1.12–1.38, *p* < 0.001)	1.32 (1.04–2.27, NS)

The table was generated using R software (version 4.3.2), and the following packages, dplyr, oddsratio broom, and epitools, were utilized. The statistical method used was binary logistic regression. The level of significance *p* value was set at 0.05. [NUPR1: Nuclear Protein 1, NDRG2: N-Myc Downstream-Regulated Gene 2, GLI1: glioma-associated oncogene homolog 1, NS: non-significant, OR: odds ratio, CI: confidence interval].

**Table 8:** Response evaluation criteria based on MRI imaging.

Patient Response	Patient Number	Brain Tumor Lesions	Assessment Criteria
CR	15	absence of all documented tumor lesions	complete absence of all previously identified enhancing lesions for a minimum duration of 4 weeks, non-enhancing lesions must remain stable or improve, patients must be off corticosteroids, and clinical stability or improvement in the patient’s overall health must be present
PR	8	50% or more reduction in precalculated tumor lesions	quantifiable enhancement of pre-assessed tumor lesions
SD	7	no changes in size of tumor lesions; a ≤50% decline	<25% increase in the size of pre-measured tumor lesions
PD	28	≥25% rise in the dimensions of some or all brain lesions	the emergence of any additional brain lesions

[CR: complete response, PR: partial response, SD: stable disease, PD: progressed disease].

**Table 9:** Discriminative, prognostic, and predictive potential of NUPR1, MGMT, NDRG2, and GL1 gene promoter methylation in GBM patients (*n* = 58).

					95% CI	
	Methylated Gene	AUC	S.E.M	Sig.	Lower Bound	Upper Bound
Discriminative potential	NUPR1	0.858	0.050	0.000	0.759	0.956
	MGMT	0.793	0.051	0.000	0.693	0.893
	NDRG2	0.973	0.018	0.000	0.939	1.000
	GLI1	0.881	0.037	0.000	0.808	0.954
Prognostic potential	NUPR1	0.802	0.060	0.000	0.684	0.920
	MGMT	0.794	0.064	0.000	0.669	0.919
	NDRG2	0.790	0.062	0.000	0.668	0.911
	GLI1	0.761	0.066	0.001	0.631	0.891
Predictive potential	NUPR1	0.832	0.057	0.000	0.721	0.943
	MGMT	0.848	0.053	0.000	0.745	0.951
	NDRG2	0.767	0.067	0.000	0.636	0.899
	GLI1	0.758	0.067	0.001	0.628	0.889

Data were obtained from the ROC curve analysis using SPSS software (version 27.0). The level of significance *p* value was set at 0.05. [MGMT: O6-methylguanine-DNA methyltransferase, NUPR1: Nuclear Protein 1, NDRG2: N-Myc Downstream-Regulated Gene 2, GLI1: glioma-associated oncogene homolog 1, AUC: area under the curve, SEM: standard error of the mean].

**Table 10:** Overall sensitivities, specificities, and predictive values for MGMT, NUPR1, NDRG2, and GLI1 gene methylation in GBM patients (*n* = 58).

Methylated Gene (Units)	MGMT	NUPR1	NDRG2	GLI1
Optimal criterion	≤19	≤43	>57	>38
AUC	0.794	0.802	0.790	0.761
SEM	0.0634	0.0606	0.0623	0.0670
*p* value	<0.0001	<0.0001	<0.0001	0.0001
95% CI	0.667–0.889	0.677–0.895	0.663–0.886	0.631–0.863
Sensitivity %	74.19	74.19	87.10	80.65
Specificity %	88.89	88.89	66.67	74.07
+LR	6.68 (2.25–19.80)	6.68 (2.25–19.80)	2.61 (1.51–4.53)	3.11 (1.61–6.02)
−LR	0.29 (0.16–0.54)	0.29 (0.16–0.54)	0.19 (0.075–0.50)	0.26 (0.12–0.55)
PPV	88.5	88.5	75	78.1
NPV	75	75	81.8	76.9
Youden index J	0.6308	0.6308	0.5376	0.5472

Data were obtained from the ROC curve analysis using MedCalc software. *p* value < 0.05. [AUC, area under the curve; PPV, positive predictive value; NPV, negative predictive value; SEM, standard error of the mean; +LR, positive likelihood ratio; −LR, negative likelihood ratio].

**Table 11:** Gene in silico characterizations (accessed 17 October 2023).

Gene	Alias	ID, Involved in	Chromosome Location, Exon, Strand
NUPR1	nuclear transcriptional regulator protein 1; protein p8 (P8); candidate of metastasis 1 (COM1)	ENSG00000176046, brain tumor	16p11.2, −, −
		https://www.encodeproject.org/genes/389493/	
MGMT	O-6-methylguanine-DNA methyltransferase; methylated–DNA–protein–cysteine methyltransferase	ENSG00000170430, cellular defense, DNA repair	10q26.3, 5, +
		https://www.encodeproject.org/genes/4255/	
NDRG2	NDRG family member 2; SYLD; KIAA1248	ENSG00000165795, tumor suppressor	14q11.2, 22, −
		https://www.encodeproject.org/genes/57447/	
GLI1	glioma-associated oncogene homolog 1 (zinc finger protein); PPD1; PAPA8; B4DNF7 (inhibited by GANT, betulinic acid)	ENSG00000111087.9, oncogene	12q13.3, 13, +
		https://www.encodeproject.org/genes/2735/	

[MGMT: O6-methylguanine-DNA methyltransferase, NUPR1: Nuclear Protein 1, NDRG2: N-Myc Downstream-Regulated Gene 2, GLI1: glioma-associated oncogene homolog 1].

**Table 12:** MGMT, NUPR1, NDRG2, and GLI1 target gene methylation status and primer sequences used for qPCR.

Target Gene	Methylation Status	Location	Amplicon Size (nt)	Primer Direction	Sequence (5′ to 3′)
MGMT	M	chr10:131273168-131282932	9764	Forward	5′-TTATTTTTGTGATAGGAAAAGGTACG-3′
				Reverse	5′-TAAAACAATCTACGCATCCTCG-3′
	U			Forward	5′-ATTTTTGTGATAGGAAAAGGTATGG-3′
				Reverse	5′-CTAAAACAATCTACACATCCTCACT-3′
NUPR1	M	chr14:97052990-97059965	6975	Forward	5′-CGGTCGAGTTAGAGTTTGTAGAC-3′
				Reverse	5′-TACTAAATCTTCCAAAAACGCC-3′
	U			Forward	5′-TGGTTGAGTTAGAGTTTGTAGATGT-3′
				Reverse	5′-ACTACTAAATCTTCCAAAAACACC-3′
NDRG2	M	chr14:23387074-23391083	4009	Forward	5′-GTTTTTGGAGTTTTAGTTTTTGTGC-3′
				Reverse	5′-GAACGATATAATTAATCCGCGTC-3′
	U			Forward	5′-TTTTGGAGTTTTAGTTTTTGTGTGT-3′
				Reverse	5′-CAAACAATATAATTAATCCACATC-3′
GLI1	M	chr12:58850870-58855746	4876	Forward	5′-ATCCTCCAGAACGGCAAGAG-3′
				Reverse	5′-GGTCCAGGGCTGGAGTCTG-3′
	U			Forward	5′-CTCATCCTCCAGAACGGCAA-3′
				Reverse	5′-AAGAGTGGGCCTCTGTCTGG-3′

Retrieved from https://www.primer3plus.com/ [MGMT: O6-methylguanine-DNA methyltransferase, NUPR1: Nuclear Protein 1, NDRG2: N-Myc Downstream-Regulated Gene 2, GLI1: glioma-associated oncogene homolog 1, M: methylated primer, U: unmethylated primer, nt: nucleotide].

## Data Availability

The datasets used during and/or analyzed during the current study are publicly available.

## Supplementary Materials

The following supporting information can be downloaded at https://www.mdpi.com/article/10.3390/ijms27020763/s1.

## Abbreviations

The following abbreviations are used in this manuscript:

ABCG8	ATP Binding Cassette Subfamily G Member 8
ACTB	Actin Beta
AKT	Protein Kinase B
AKT2	AKT Serine/Threonine Kinase 2
AKT3	AKT Serine/Threonine Kinase 3
ALDH1A1	Aldehyde Dehydrogenase 1 Family Member A1
ANP32E	Acidic Nuclear Phosphoprotein 32 Family Member E
ANXA1	Annexin A1
ANXA2	Annexin A2
ARRB2	Beta-Arrestin-2
ATP1B1	ATPase Na+/K+ Transporting Subunit Beta 1
AUC	Area Under the Curve
BCL2	B-Cell Leukemia/Lymphoma 2 Protein
BMP2	Bone Morphogenetic Protein 2
BMP4	Bone Morphogenetic Protein 4
BMP5	Bone Morphogenetic Protein 5
BMP6	Bone Morphogenetic Protein 6
BMP7	Bone Morphogenetic Protein 7
BMP8A	Bone Morphogenetic Protein 8a
BMP8B	Bone Morphogenetic Protein 8b
BRCA2	BRCA2 DNA Repair Associated
BRIP1	BRCA1 Interacting Protein C-Terminal Helicase 1
BTRC	Beta-Transducin Repeat-Containing Protein
BTRC	Beta-Transducin Repeat-Containing E3 Ubiquitin Protein Ligase
CBG	Capacity Building Grant Fund
CCNB1	Cyclin B1
CDK1	Cyclin-Dependent Kinase 1
CDK2	Cyclin-Dependent Kinase 2
CDK4	Cyclin-Dependent Kinase 4
CDK4/6	Cyclin-Dependent Kinase 4/6
CDK5	Cyclin-Dependent Kinase 5
CDK6	Cyclin-Dependent Kinase 6
CDK10	Cyclin-Dependent Kinase 10
CDK12	Cyclin-Dependent Kinase 12
CDKN2A	Cyclin-Dependent Kinase Inhibitor 2A
CISH	Cytokine Inducible SH2 Containing Protein
CLN8	Ceroid Lipofuscinosis Neuronal 8
CNS	Central Nervous System
COPS5	COP9 Signalosome Subunit 5
CR	Complete Response
CREBBP	CREB-Binding Protein
CSNK1A1	Casein Kinase 1 Alpha 1
CSNK1A1L	Casein Kinase 1 Alpha 1 like
CSNK2A1	Casein Kinase 2 Alpha 1
CSNK2A2	Casein Kinase 2 Alpha 2
CTDP1	CTD Phosphatase Subunit 1
DEAD-Box Helicase 5	DDDX5
DEAD-Box Helicase 24	DDDX24
DNMT1	DNA Methyltransferase 1
E2F1/3	E2F Transcription Factors 1/3
ECOG	Ester Clinical Oncology Group
EEF1A1	Eukaryotic Translation Elongation Factor 1 Alpha 1
EGFR	Epidermal Growth Factor Receptor
EP300	E1A Binding Protein P300
ESR1	Estrogen Receptor 1
FFPE	Formalin-Fixed Paraffin-Embedded
FXR1	Fragile X Mental Retardation 1
FXR2	FMR1 Autosomal Homolog 2
GBM	Glioblastoma
GLI1	Glioma-Associated Oncogene Homolog 1
GLI2	Glioma-Associated Oncogene Homolog 2
GLI3	Glioma-Associated Oncogene Homolog 3
GTR	Gross Total Resection
Gy	Gray (unit of absorbed radiation dose)
Gd-MRI	Gadolinium-Enhanced Magnetic Resonance Imaging
HAX1	HCLS1-Associated Protein X 1
HE	Hematoxylin–Eosin (staining method for histology)
HH	Hedgehog Pathway
HIF1A	Hypoxia Inducible Factor 1 Subunit Alpha
IDH1	Isocitrate Dehydrogenase (NADP(+)) 1
IDH2	Isocitrate Dehydrogenase (NADP(+)) 2
IHH	Indian Hedgehog
INS	Insulin
IQR	Interquartile Range
KIF7	Kinesin Family Member 7
KRAS	Kirsten Rat Sarcoma Viral Oncogene Homolog
KWt	Kruskal–Wallis H Test
LR	Likelihood Ratio
+LR	Positive Likelihood Ratio
-LR	Negative Likelihood Ratio
MDM2	MDM2 Proto-Oncogene
MGMT	O-6-Methylguanine-DNA Methyltransferase
MLH1	MutL Homolog 1
MMP2	Matrix Metallopeptidase 2
MMP9	Matrix Metallopeptidase 9
MOB2	MOB Kinase Activator 2
MPG	Membrane-Bound O-Acyltransferase Domain Containing 2
MRI	Magnetic Resonance Imaging
MSH2	MutS Homolog 2
MSH3	MutS Homolog 3
MSH6	MutS Homolog 6
mTOR	Mechanistic Target of Rapamycin
MVP	Microvascular Proliferation
MYCN	MYCN Proto-Oncogene, BHLH Transcription Factor
MYOD1	Myogenic Differentiation 1
NDRG2	N-Myc Downstream-Regulated 2
NND	Non-Neurooncological Diseases
NPV	Negative Predictive Value
NS	Non-Significant
NUPR1	Nuclear Protein 1
NPY5R	Neuropetide Y Receptor Y5
OR	Odds Ratio
OS	Overall Survival
PAXIP1	PAX Interacting Protein 1
PD	Progressive Disease
PDGF	Platelet-Derived Growth Factor
PDGFR	Platelet-Derived Growth Factor Receptor
PFS	Progression-Free Survival
PI3K	Phosphoinositide 3-Kinase
PMS2	PMS2 Postmeiotic Segregation Increased 2 (S. cerevisiae)
PR	Partial Response
PRKACA	Protein Kinase cAMP-Activated Catalytic Subunit Alpha
PTCH1	Patched 1
PTEN	Phosphatase and Tensin Homolog
PTMA	Prothymosin, Alpha
p53	Tumor Protein p53
qPCR	Quantitative Polymerase Chain Reaction
RAB4C1	RAB4C, Member RAS Oncogene Family
RANO	Response Assessment in Neuro-Oncology
RASSF1	Ras Association Domain Family Member 1
RB	Retinoblastoma
RB1	Retinoblastoma Protein
ROC	Receiver Operating Characteristic
ROS	Reactive Oxygen Species
RTK/Ras/PI3K	Receptor Tyrosine Kinase/Ras/Phosphoinositide 3-Kinase
S.D	Standard Deviation
SD	Stable Disease
SEM	Standard Error of the Mean
SET	SET Nuclear Proto-Oncogene
SHH	Sonic Hedgehog
SMO	Smoothened, Frizzled Family Receptor
SN	Sensitivity
SOX2	SRY (Sex-Determining Region Y)-Box 2
SP	Specificity
SPOP	Speckle Type POZ Protein
SPSS	Statistical Package for Social Studies Software
SR	Subtotal Resection
SST	Somatostatin
STDF	Science, Technology & Innovation Funding Authority
STK36	Serine/Threonine Kinase 36
STK38L	Serine/Threonine Kinase 38 Like
SUFU	Suppressor Of Fused Homolog (Drosophila)
T.R.	Therapeutic Response
TCF12	Transcription Factor 12
TCF15	Transcription Factor 15
TCF19	Transcription Factor 19
TCF20	Transcription Factor 20
TCF23	Transcription Factor 23
TMZ	Temozolomide
TP53	Tumor Protein P53
TSC1/2	Tuberous Sclerosis Complex 1/2
U	Unmethylated Primer
VEGF	Vascular Endothelial Growth Factor
